# Redox-Active Metal-Organic Frameworks with Three-Dimensional Lattice Containing the *m*-Tetrathiafulvalene-Tetrabenzoate

**DOI:** 10.3390/molecules27134052

**Published:** 2022-06-23

**Authors:** Hongrui Huang, Zhi-Mei Yang, Xiao-Cheng Zhou, Gen Zhang, Jian Su

**Affiliations:** 1 School of Chemistry and Chemical Engineering, Nanjing University of Science and Technology, Nanjing 210094, China; hhrvinc@njust.edu.cn; 2 State Key Laboratory of Coordination Chemistry, Nanjing University, Nanjing 210023, China; dg21240145@smail.nju.edu.cn (Z.-M.Y.); mg20240142@smail.nju.edu.cn (X.-C.Z.)

**Keywords:** metal-organic framework, lanthanide, redox activity, tetrathiafulvalene

## Abstract

Metal-organic frameworks (MOFs) constructed by tetrathiafulvalene-tetrabenzoate (H_4_TTFTB) have been widely studied in porous materials, while the studies of other TTFTB derivatives are rare. Herein, the meta derivative of the frequently used *p*-H_4_TTFTB ligand, *m*-H_4_TTFTB, and lanthanide (Ln) metal ions (Tb^3+^, Er^3+^, and Gd^3+^) were assembled into three novel MOFs. Compared with the reported porous Ln-TTFTB, the resulted three-dimensional frameworks, Ln-*m*-TTFTB ([Ln_2_(*m*-TTFTB)(*m*-H_2_TTFTB)_0.5_(HCOO)(DMF)]·2DMF·3H_2_O), possess a more dense stacking which leads to scarce porosity. The solid-state cyclic voltammetry studies revealed that these MOFs show similar redox activity with two reversible one-electron processes at 0.21 and 0.48 V (vs. Fc/Fc^+^). The results of magnetic properties suggested Dy-*m*-TTFTB and Er-*m*-TTFTB exhibit slow relaxation of the magnetization. Porosity was not found in these materials, which is probably due to the meta-configuration of the *m*-TTFTB ligand that seems to hinder the formation of pores. However, the *m*-TTFTB ligand has shown to be promising to construct redox-active or electrically conductive MOFs in future work.

## 1. Introduction

In the development of functional metal-organic frameworks (MOFs), MOFs with special reactivity are highly demanded. Redox-active MOFs with good stimuli response have shown potential applications in intelligent materials [1,2,3,4]. Focusing on the components of MOFs, the redox activity could arise from variable metal centers, redox-active organic linkers, and/or redox-active guest molecules confined in the cages or channels in the framework. The modulation of different redox states which show distinct physical properties can be achieved via post-synthetic redox reactions or electrochemical methods [5,6,7,8,9]. In various organic building blocks, the electron-rich tetrathiafulvalene (TTF) unit, owing to its two stable oxidized states, has become a famous redox-active building block in the construction of functional MOFs [1,10]. Among the TTF-carboxylate ligands, dimethylthio-tetrathiafulvalene-bicarboxylate (H_2_TTFBC), tetrathiafulvalene-tetracarboxylate (H_4_TTFTC), and tetrathiafulvalene-tetrabenzoate (H_4_TTFTB) are well-researched in the previous works [10,11,12,13,14,15,16,17]. For H_4_TTFTB, Dincă et al. first reported its synthesis and assembly of Zn^2+^ in a semiconductive MOF [18]. Since then, the self-assembly of H_4_TTFTB with diverse metal ions including Cd^2+^, Mn^2+^, Co^2+^, Ba^2+^, Fe^2+/3+^, Mg^2+^, In^3+^, and Zr^4+^ has been successfully realized [6,19,20,21,22,23,24,25,26]. _ENREF_15 In addition, the combination of H_4_TTFTB and lanthanide (Ln) ions has resulted in diverse two dimensional or three-dimensional MOFs [27,28,29,30]. For example, in 2019, Dincă et al. reported three polymorphic MOFs containing La^3+^ and TTFTB. These MOFs crystallize with unique topologies and exhibit different intermolecular π···π stacking interactions within the TTF moieties [29]. In summary, owing to the large angle between adjacent carboxylates in H_4_TTFTB, TTFTB-based MOFs always show good porosity.

Comparatively speaking, the meta-derivative of the frequently used *p*-TTFTB ligand is rarely reported. It can be predicted that H_4_TTFTB will show a different assembly behavior in the construction of MOFs. In 2020, Zuo et al. firstly reported a 2D MOF, In-*m*-TTFTB, which possesses a proton conductivity of 6.66 × 10^−4^ S cm^−1^ at 303 K and 98% relative humidity (RH) [31]. Recently, a series of persistent radical 2D MOFs were assembled by a hexanuclear rare-earth-cluster-based 1D chains and a (*m*-TTFTB)_3_ trimer building block [32]. These MOFs exhibit highly chemical and thermal stability. Due to efficient light absorption, intermolecular charge transfer, low thermal conductivity, and outstanding stability, Dy_6_-*m*-TTFTB-MOF shows excellent photothermal properties, an increase of 34.7 °C within 240 s under one-sun illumination [32]. Another kind of *m*-TTFTB-MOF was obtained by adjusting the synthetic conditions. This MOF possesses a low BET surface area of 129 m^2·^g^−1^ with a high near-infrared (NIR) photothermal conversion [33]. Further study revealed that the photothermal conversion of this MOF could be enhanced by redox doping and plasmon resonance. Even though the photothermal conversion of *m*-TTFTB-based MOFs is carefully studied [31,32], the semiconductive and magnetic properties of these MOFs have not been reached.

**Scheme 1:**
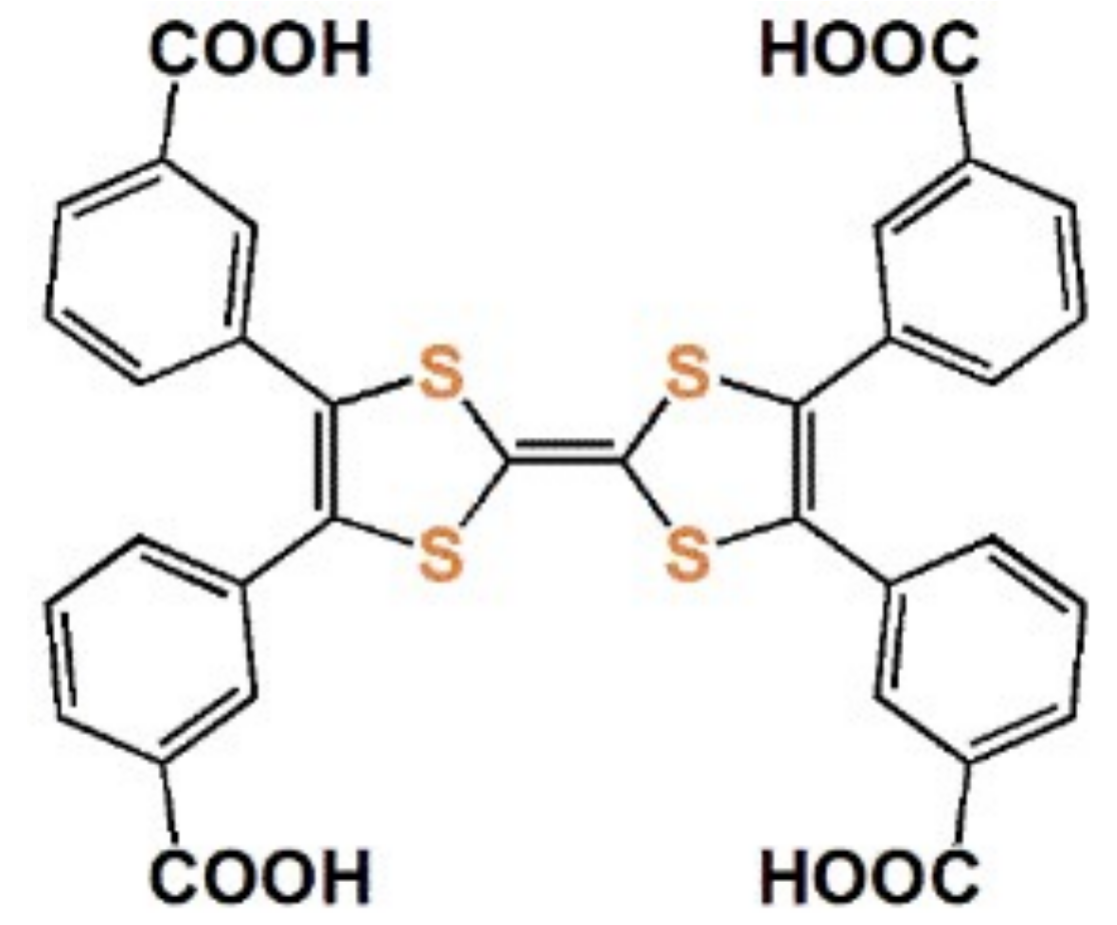
Molecular structure of the *m*-H_4_TTFTB ligand.

The present work reported three MOFs ([Ln_2_(*m*-TTFTB)(*m*-H_2_TTFTB)_0.5_(HCOO)(DMF)]·2DMF·3H_2_O) generated from *m*-TTFTB (Scheme 1) and lanthanide ions (Ln = Tb^3+^, Er^3+^, and Gd^3+^) using solvothermal methods. The framework consists of an improved inorganic component, a chain of Ln metals bound by carboxylates with a dense stacking. All the MOFs were studied by single-crystal X-ray diffraction structural characterizations and their redox activities, light absorption, electrical conductivities, and magnetic properties are discussed.

## 2. Results and Discussion

### 2.1. Crystal Structures

**Figure 1:**
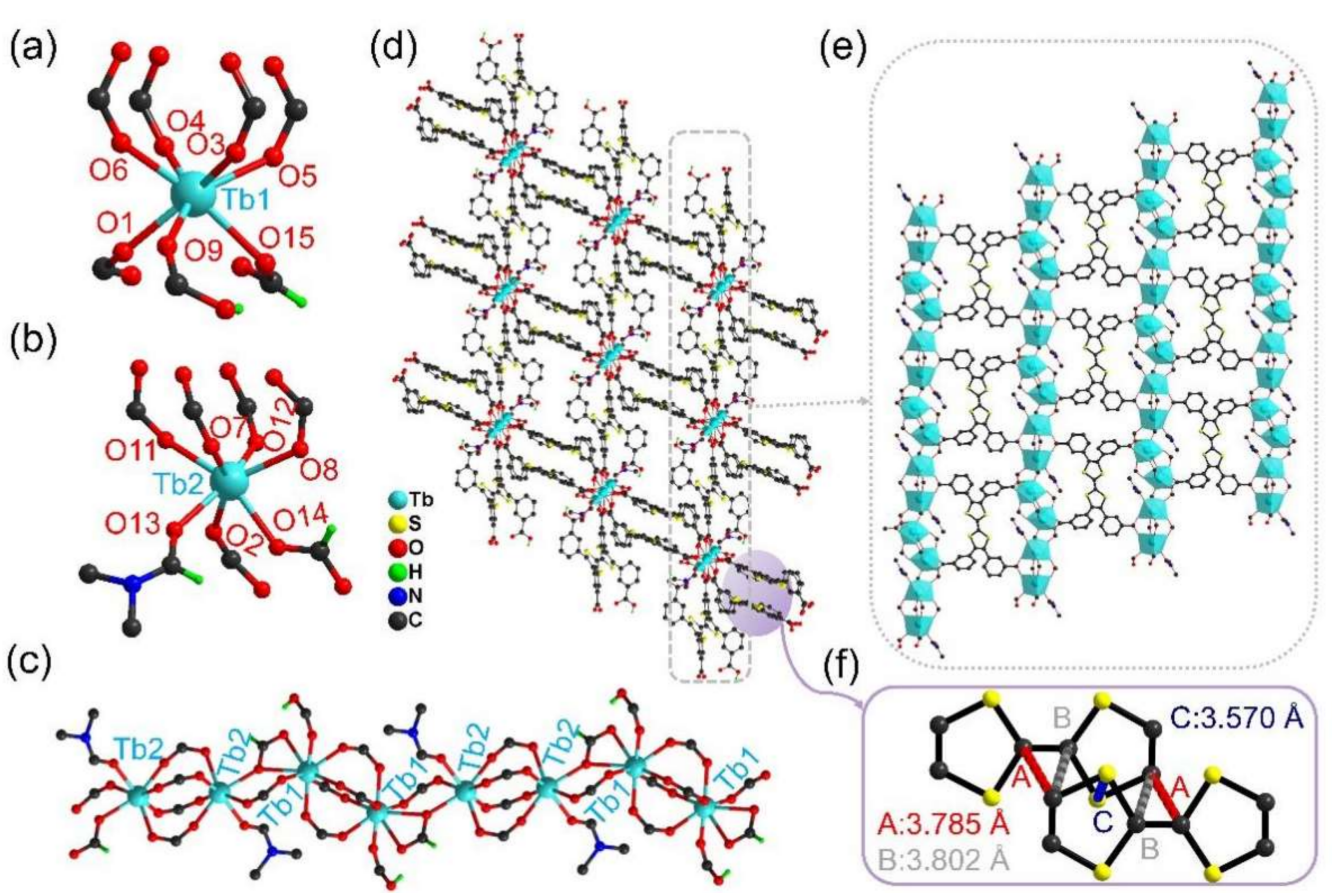
Structure of Tb-*m*-TTFTB ([Tb_2_(*m*-TTFTB)(*m*-H_2_TTFTB)_0.5_(HCOO)(DMF)]·2DMF·3H_2_O): the coordination environments of Tb1 (**a**) and Tb2 (**b**); a one-dimensional chain in Tb-*m*-TTFTB (**c**); (**d**) view of the three-dimensional framework along the [111] direction; (**e**) the two-dimensional structure assembled by the 1D chains and *m*-TTFTB^4−^ ligands; (**f**) the weak interaction between adjacent TTF linkers. Some of the *m*-TTFTB^4−^ ligands are simplified to benzoate group for clarity.

Analyses of the diffraction data for Tb-*m*-TTFTB, Er-*m*-TTFTB, and Gd-*m*-TTFTB (Figure S1) revealed that they are isostructural three-dimensional MOFs crystallizing in the triclinic space group *P-1* (Table 1). For Tb-*m*-TTFTB, two kinds of crystallographic-independent Tb^3+^ ions and *m*-TTFTB ligands were observed, respectively (Figure S2). The coordination numbers of Tb1 and Tb2 atoms were both seven (Figure 1a,b). One of the *m*-TTFTB^4−^ (L1) ligands was coordinated to eight Tb atoms (Figure S3). Another *m*-H_2_TTFTB^2−^ (L2) was coordinated to six Tb atoms, and half of the carboxylates were in the protonated state (Figure S3). The Tb−O bond lengths were in the range of 2.266 to 2.478 Å (Table S1), which are comparable to those reported for Tb-MOFs [30,34]. The Tb1 pairs and Tb2 pairs linked by two *anti–anti* carboxylates (Tb1···Tb2 = 4.70 Å) form the one-dimensional structure (Figure 1c). The Tb pairs both have four bridging *syn*–*syn* carboxylates (four-blade paddle-wheel; Tb1···Tb1 = 4.39 Å, Tb2···Tb2 = 4.20 Å). In the reported structural parameters of Tb-TTFTB, the distances of Tb1···Tb1, Tb2···Tb2, and Tb1···Tb2 were 4.56, 4.21, and 5.47 Å, respectively [34]. In the chain structure of Tb-*m*-TTFTB, the increased number of binding carboxylates between Tb1···Tb1 and Tb1···Tb2 compared with Tb-TTFTB enhanced the interaction of the Tb centers and thus reduced the relative distance. Each one-dimensional chain is linked by the *m*-TTFTB^4−^ (Figure 1e) to generate the two-dimensional network. The weak interactions of S···S (3.57 Å) and C···C (3.79 Å) in Tb-*m*-TTFTB, S···S (3.50 Å) and C···C (3.74 Å) in Er-*m*-TTFTB, and S···S (3.56 Å) and C···C (3.74 Å) in Gd-*m*-TTFTB were observed (Figure 1f). Notably, these S···S distances between the TTF linkers are even smaller than the distance in the conducting framework of Cd_2_(TTFTB) (3.65 Å) [19]. Finally, the three-dimensional framework of Tb-*m*-TTFTB (Figure 1d) can be assembled by the 2D plane in Figure 1e and interacted *m*-TTFTB^4−^. The three-dimensional structures along three unit cell axes showed non-porosity (Figure S4).

**Figure 2:**
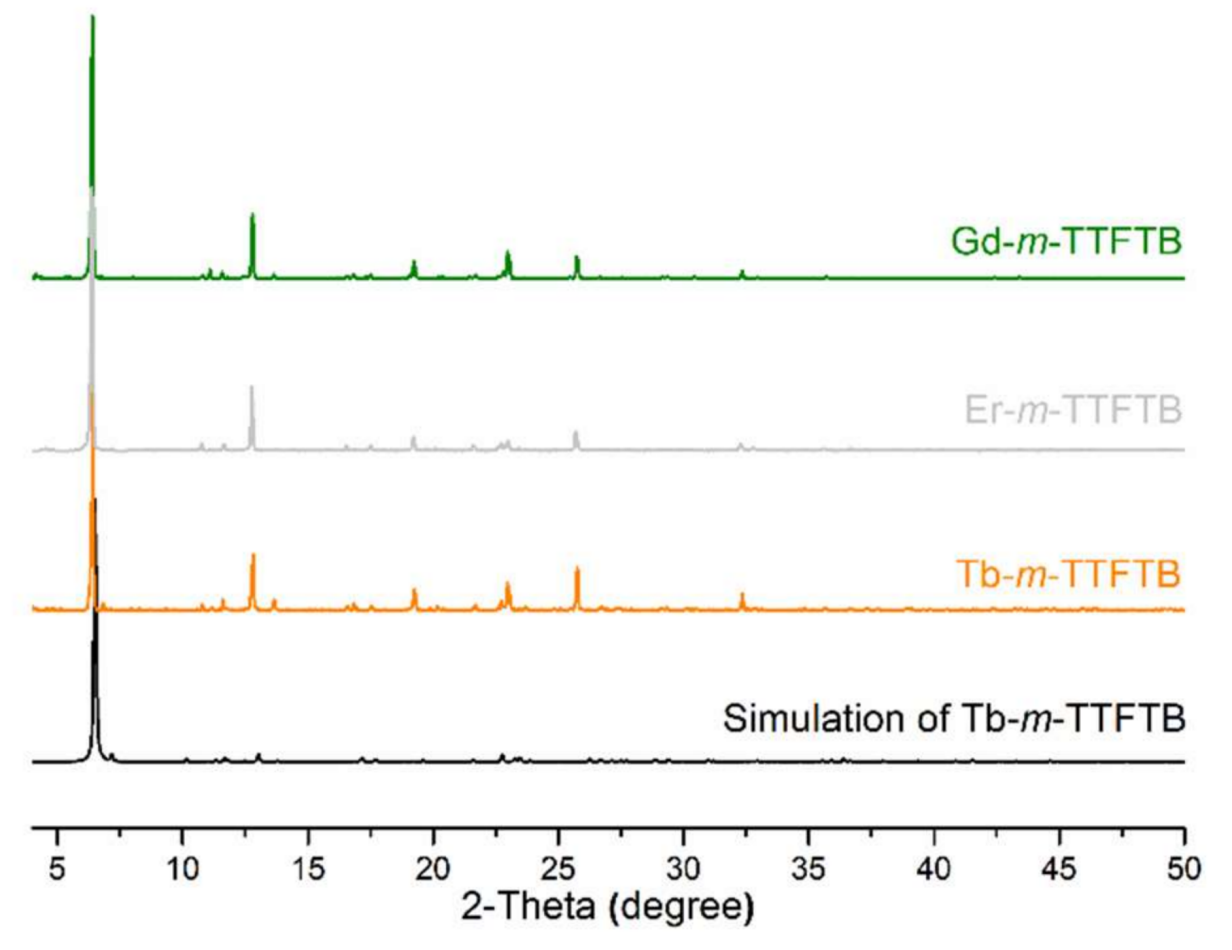
Experimental and calculated powder X-ray diffraction patterns for Tb-*m*-TTFTB, Er-*m*-TTFTB, and Gd-*m*-TTFTB. PXRD was simulated based on the single-crystal structure of Tb-*m*-TTFTB by applying preferred orientation along the *b*-direction.

The absence of any other phases from Tb-*m*-TTFTB, Er-*m*-TTFTB, and Gd-*m*-TTFTB was confirmed by powder X-ray diffraction (PXRD) measurements in which the diffraction peak positions were similar to the calculated from single-crystal X-ray data of Tb-*m*-TTFTB (Figure 2). The advantages in stability originated from the interaction of TTF matrix and the protecting of the tight organic parts surrounding the rare earth centers (Figure 1c) [33]. In this case, reducing of coordination space is an efficient strategy to enhance the stability of the frameworks. The thermal stability was investigated. The TGA data for Ln-*m*-TTFTB showed that the frameworks have good thermal stability up to 450 °C (Figure S5) in the N_2_ atmosphere, which is comparable to the Ln-TTFTB series (nearly 500 °C) [28,34].

### 2.2. Cyclic Voltammetry

**Figure 3:**
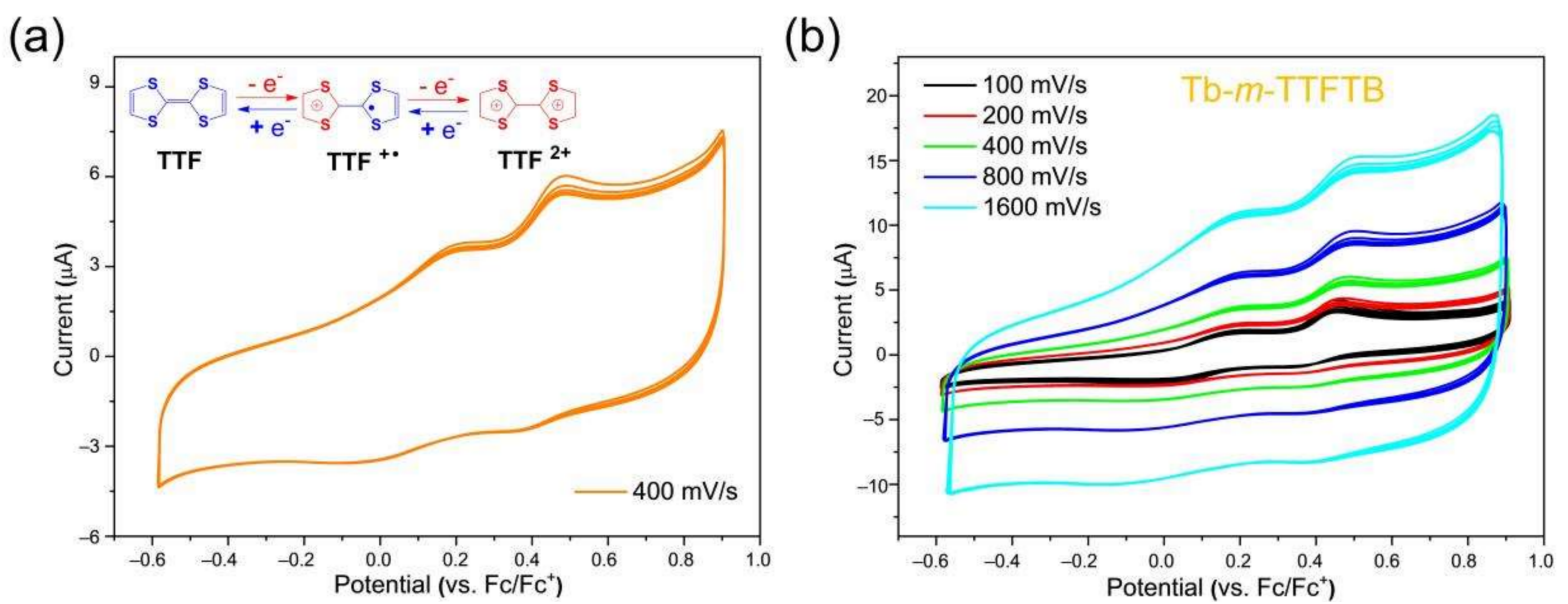
Solid-state cyclic voltammograms of Tb-*m*-TTFTB performed over five consecutive cycles (**a**) and at different scan rates (**b**). The experiments were conducted in 0.1 M LiBF_4_ in CH_3_CN electrolyte. The inset is the redox reaction of the TTF.

Solid-state direct current (DC) cyclic voltammetry (CV) studies on Ln-*m*-TTFTB were conducted in 0.1 M LiBF_4_ in CH_3_CN (Figure 3a and Figures S6 and S7). Upon scanning anodically, two reversible one-electron processes at around 0.21 and 0.48 V (vs. Fc/Fc^+^) were observed for all three MOFs. These processes are attributed to the TTF/TTF^•+^ and TTF^•+^/TTF^2+^ redox couples, respectively (inset of Figure 3a). In contrast to the CV of *m*-H_4_TTFTB (0.13 and 0.35 V (vs. Fc/Fc^+^)) [31], the two one-electron processes observed for Tb-*m*-TTFTB were shifted by ca. 0.1 V, which is attributed to both the coordination to terbium ions and the deprotonated nature of the ligand [35]. The current associated with the two redox processes were almost the same over multiple scans which is consistent with their reversible nature. In addition, faster sweep rates led to broader features because of slow diffusion kinetics through the framework (Figure 3b and Figures S6 and S7). The locations of these redox couples in Ln-*m*-TTFTB were similar to those of Ln-TTFTB, meaning that these two series of MOFs possess similar redox activity [34].

### 2.3. Absorption Spectra and Semiconducting Properties

**Figure 4:**
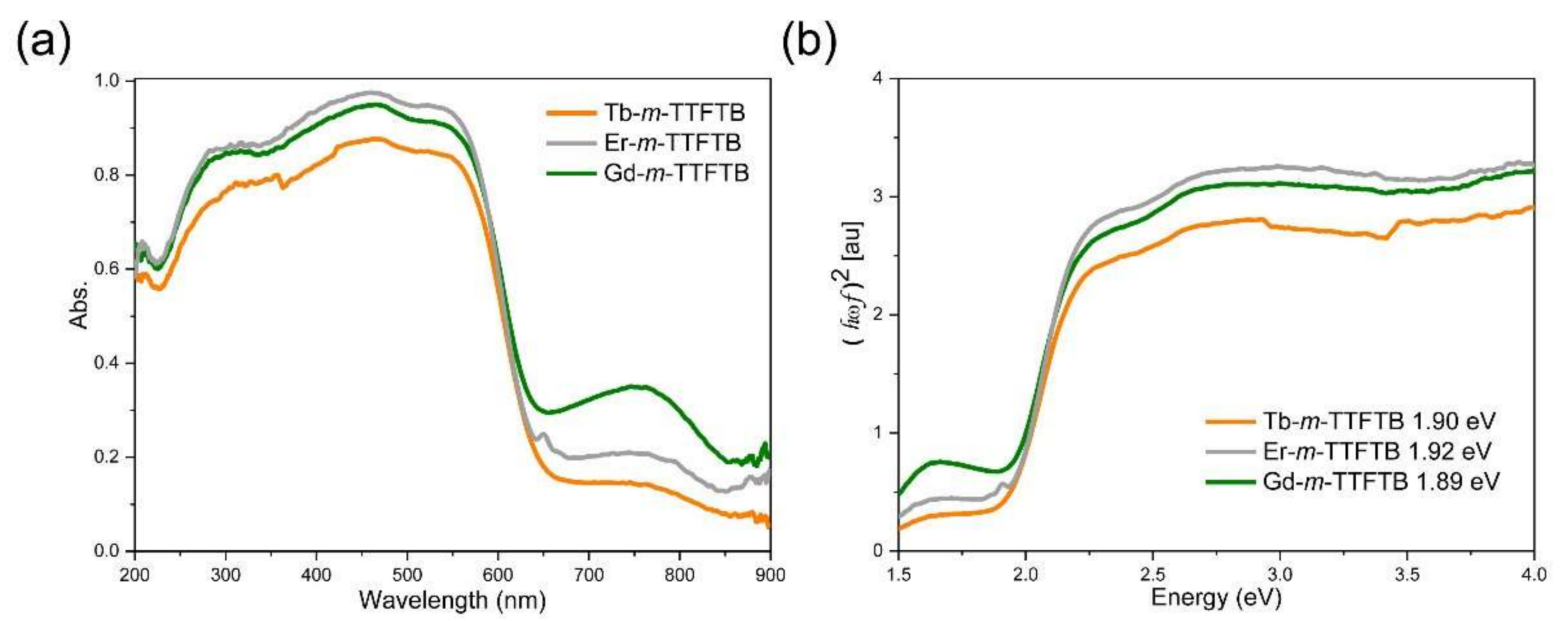
The diffuse reflectance spectra (**a**) and the translated Tauc plots (**b**) for Tb-*m*-TTFTB, Er-*m*-TTFTB, and Gd-*m*-TTFTB.

Prior to studying the conductivities of these materials, the solid-state absorption spectra were obtained to gain insight into the influence of coordination on the optical and conducting properties. In Ln-*m*-TTFTB, there are three main absorption bands located in the region 250–550 nm (Figure 4a). These higher energy absorption bands are attributed to the n→π* or π→π* transition of the free ligand, which is similar to the absorption of the ligand *m*-H_4_TTFTB [33]. The absorption band around 760 nm can be assigned to the neutral (TTF)_2_ in the framework [36,37]. The peak located at 690 nm of the free ligand can be attributed to a small number of auto-oxidized TTF^•+^. Using these UV-vis-NIR adsorption data, we approximated the band gaps of the ligand and Ln-*m*-TTFTB through the Kubelka–Munk function. From the Tauc plots [38], the band gaps of *m*-H_4_TTFTB, Gd-*m*-TTFTB, Tb-*m*-TTFTB, and Er-*m*-TTFTB were approximately 1.87, 1.89, 1.90, and 1.92 eV, respectively (Figure 4b). In general, these values are similar to the Ln-TTFTB series. This result indicated that the different locations of the carboxyl groups have a slight influence in the band gap.

To better understand the conducting behaviors of these MOFs, conductivity studies were undertaken on the single crystal samples of Ln-*m*-TTFTB by the two-contact probe method. The room temperature electrical conductivities in the long horizontal direction of the single crystal (Figure S8) were 5.4 × 10^−7^, 9.6 × 10^−7^, and 1.0 × 10^−7^ S·cm^−1^ for Tb-*m*-TTFTB, Er-*m*-TTFTB, and Gd-*m*-TTFTB, respectively (Figures S9–S11 and Table S2)_._ These values are nearly ten times of the reported Ln-TTFTB series (10^−8^ S·cm^−1^ in powder state), and this can be attributed to the lower contact resistance in single crystal (also ~10^−8^ S·cm^−1^ in powder state). The lower conducting performances of Ln-*m*-TTFTB originate from the lack of band formation or the poor electron transfer. Notably, the powder electrical conductivity is reported as 2.74 × 10^−8^ S·cm^−1^ for the free *m*-H_4_TTFTB [31]. The similar electrical conductivity between the free ligand and the assembled MOFs are predicted by the similar band gaps.

### 2.4. Magnetic Properties

**Figure 5:**
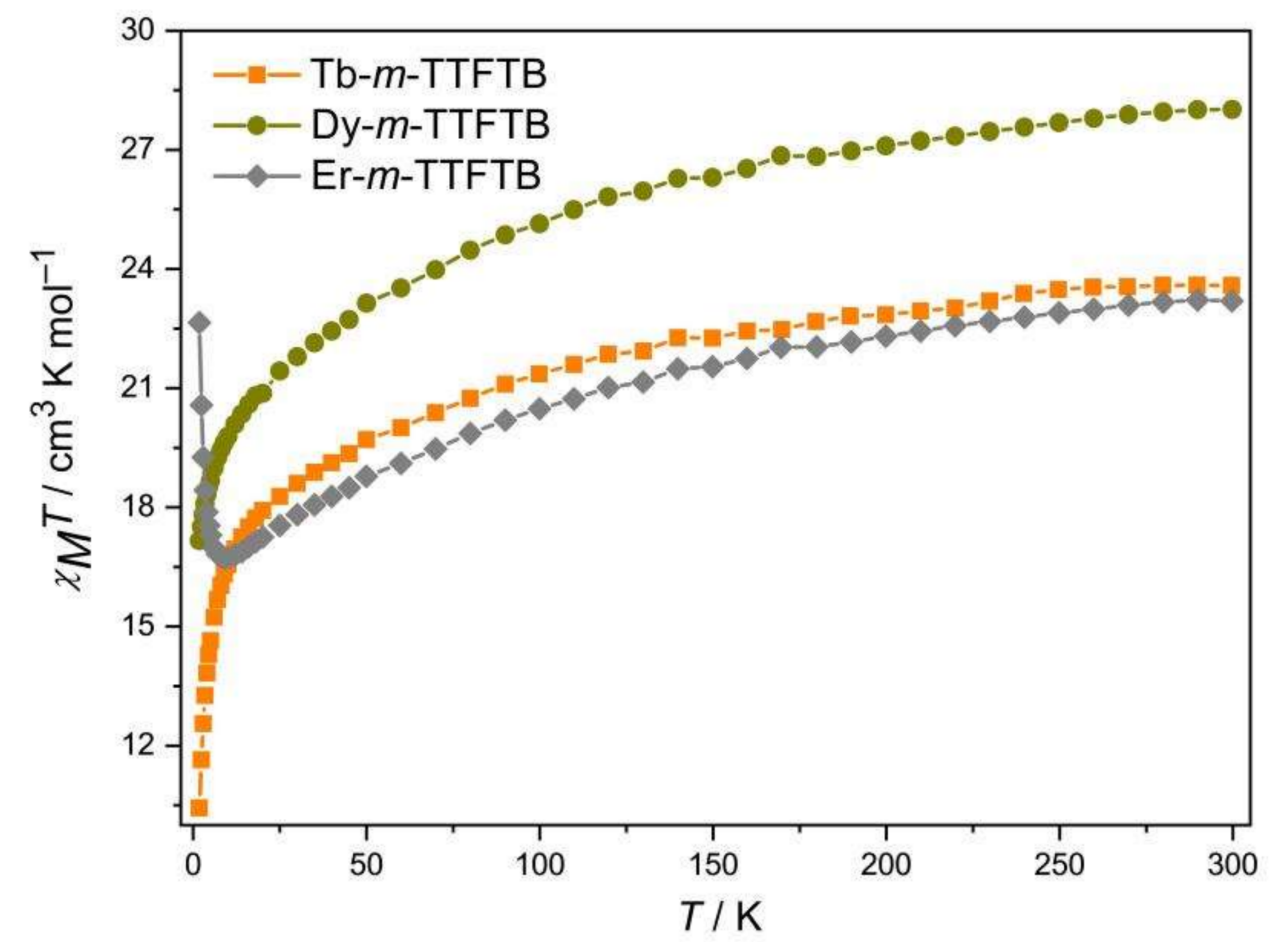
Temperature dependence of the *χ*_M_*T* measured in a 1000 Oe field.

Magnetic susceptibility measurements on fresh polycrystalline samples of Tb-*m*-TTFTB, Dy-*m*-TTFTB, and Er-*m*-TTFTB were performed in the temperature range 2–300 K under an applied magnetic field of 1000 Oe. Their *χ*_M_*T* values at 300 K were 23.59, 28.01, and 23.22 cm^3^·K·mol^−1^ for Tb-*m*-TTFTB, Dy-*m*-TTFTB, and Er-*m*-TTFTB, respectively (Figure 5). These values are consistent with theoretical values for two independent Ln^3+^ ions, 23.64 cm^3^·K·mol^−1^ (Tb^3+^, ^7^*F*_6_, *J* = 6, *g* = 3/2), 28.34 cm^3^·K·mol^−1^ (Dy^3+^, ^6^*H*_15/2_, *J* = 15/2, *g* = 4/3), and 22.95 cm^3^·K·mol^−1^ (Er^3+^, ^4^*I*_15/2_, *J* = 15/2, *g* = 6/5) [34]. As the temperature was lowered, *χ*_M_*T* decreased in each case as expected from antiferromagnetic interaction and/or the depopulation of excited states [39,40]. Focusing on the crystal structures, we can define three exchange interactions (Figure S12) within one chain due to the three different kinds of lanthanide dimer. Given that the Gd^3+^ ion is magnetically isotropic, the *χ*_M_*T* of Gd-*m*-TTFTB (Figure S13) decreased in the high-temperature region due to the antiferromagnetic exchange interaction with *J* = −1 cm^−1^ (${\hat{H}}_{EX}=-2J_{1}S_{Gd1}S_{Gd2}$) in dimers. In the low-temperature region, the slight upturn might be attributed to the ferromagnetic interaction and/or dipolar–dipolar interaction between dimers, or ferromagnetic intra-chain interaction. For Er-*m*-TTFTB, an upturn of the moment below ca. 6 K indicates one possible weak nearest neighbor ferromagnetic interaction and/or dipolar–dipolar interaction [41]. At 1.8 K, the isothermal magnetizations (*M*) versus field (*H*) reached 10.84, 11.71, and 11.08 *Nβ* in 70 kOe for Tb-*m*-TTFTB, Dy-*m*-TTFTB, and Er-*m*-TTFTB, respectively (Figures S14–S16). The low saturation of magnetization values maybe ascribed to the effects of low-lying excited states and/or magnetic anisotropy [42].

**Figure 6:**
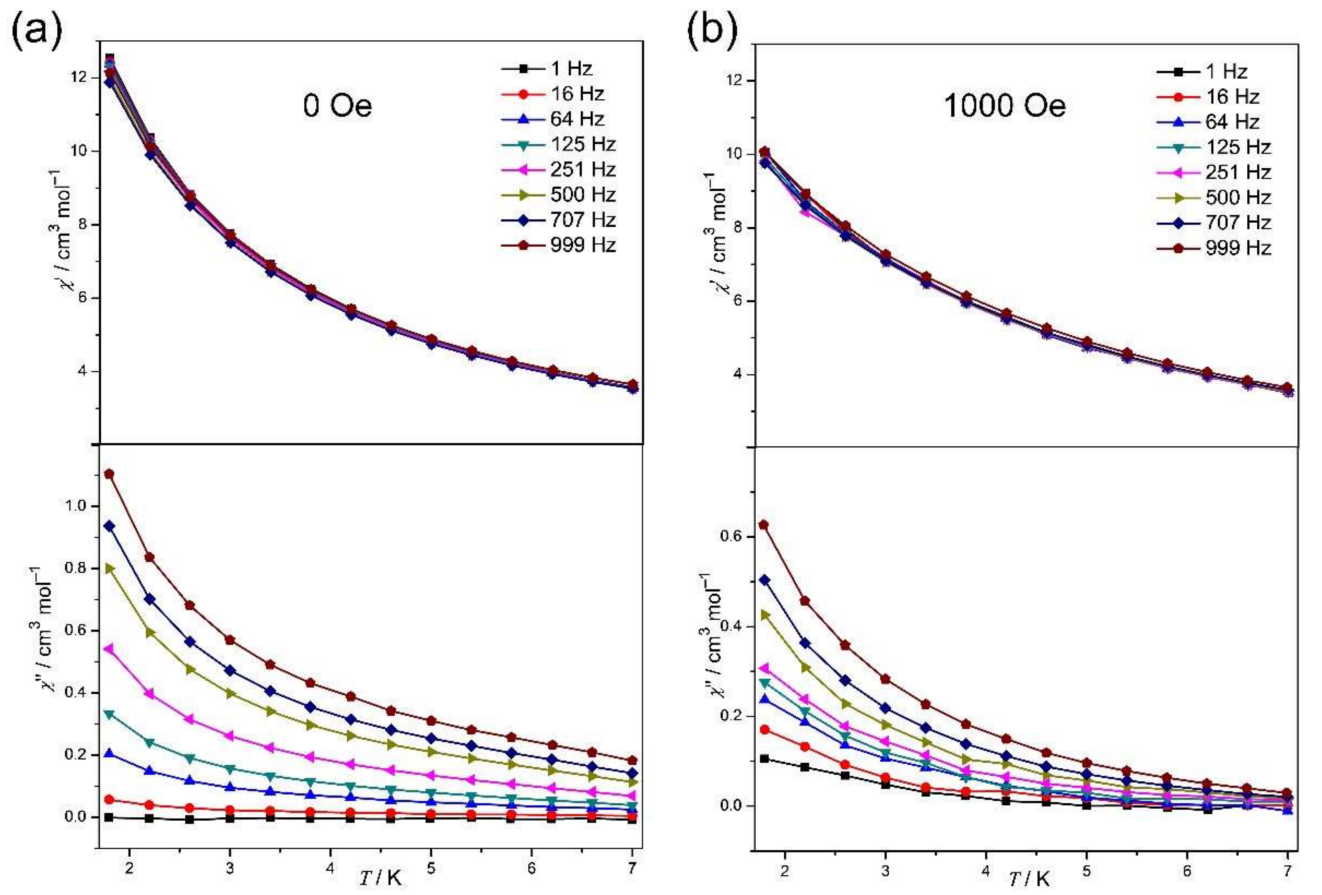
(**a**) Temperature-dependent in-phase χ′ and out-of-phase χ″ ac susceptibility signals for Dy-*m*-TTFTB at the indicated frequencies under zero dc field. (**b**) Temperature-dependent in-phase χ′ and out-of-phase χ″ ac susceptibility signals for Dy-*m*-TTFTB at the indicated frequencies under 1000 Oe.

The temperature dependence of the alternating current (ac) susceptibilities (2 Oe) at different fixed frequencies (1.0–999 Hz) were measured for all MOFs. Under zero direct current (dc) field, Dy-*m*-TTFTB exhibited both in-phase (*χ*′) and out-of-phase (*χ*″) ac-susceptibilities (Figure 6a), but no peaks were observed. The ac-susceptibilities of Er-*m*-TTFTB and Tb-*m*-TTFTB exhibited no *χ*″ in zero dc field (Figures S17a and S18a). The frequency-dependent out-of-phase (*χ*″) ac-susceptibility below 6 K for Dy-*m*-TTFTB revealed the slow relaxation of the magnetization. However, no peak of *χ*″ was observed even at 999 Hz, likely due to a lower anisotropic energy barrier. The *χ*″ susceptibility increased with the increase of the frequency, suggesting that the peak maxima are to be found at lower temperatures or higher frequencies of the SQUID instrument. Even an increase in the dc field to 1000 Oe showed no peak above 2 K in the ac-susceptibilities of Dy-*m*-TTFTB and Er-*m*-TTFTB, while Tb-*m*-TTFTB exhibited no *χ*″ signals (Figure 6b, Figures S17b and S18b). With low energy barriers, no peaks above 1.8 K in the out-of-phase of ac susceptibility observed in the frequency region of 1.0–999 Hz were reasonable.

## 3. Materials and Methods

### 3.1. Materials and Methods

All the reagents and solvents were commercially available and used as received. FT-IR spectra were recorded on a Vector 27 Bruker Spectrophotometer by transmission through KBr pellets containing the ground crystals in the range 4000–400 cm^−1^. The powder X-ray diffraction patterns (PXRD) were collected at room temperature using a scan speed of 0.1 s/step on a Bruker Advance D8 diffractometer (40 kV, 40 mA) (Bruker, Karlsruhe, Germany) equipped with Cu radiation. Calculated PXRD patterns were generated using Mercury 3.0 [43]. Elemental analyses (EA) for C, H, and N were performed on a Perkin-Elmer 240C analyzer (PerkinElmer, Waltham, MA, USA). TGA data were obtained on a STA 449C thermal analysis system at a heating rate of 10 °C min^−1^ under N_2_ atmosphere. Magnetization measurements were performed using a Quantum Design SQUID VSM magnetometer (Quantum Design, Darmstadt, Germany) on polycrystalline samples for all compounds.

### 3.2. X-ray Structure

Single-crystal X-ray diffraction intensity data for Tb-*m*-TTFTB, Er-*m*-TTFTB, and Gd-*m*-TTFTB were collected on a Bruker D8 Venture diffractometer fitted with a PHOTON-100 CMOS detector, monochromatized microfocus Mo K_α_ radiation (λ = 0.71073 Å), and a nitrogen flow controlled by a KRYOFLEX II low-temperature attachment operating at 153 K. Raw data collection and reduction were controlled using APEX3 software (version 2016.9-0; Bruker, 2016) [44]. Absorption corrections were applied using the SADABS routine. The structures were solved by direct methods and refined by full-matrix least-squares on *F*^2^ using the SHELXTL software package (version-2018/3) [45]. Non-hydrogen atoms were refined with anisotropic displacement parameters during the final cycles. Hydrogen atoms of *m*-TTFTB, formate, and dimethylformamide (DMF) molecules were placed at calculated ideal positions and isotropic displacement parameters were used. Except for the coordinated DMF molecule, those free solvent molecules of DMF or water were highly disordered and were unable to be located and refined. The diffuse electron densities resulting from these residual molecules were removed from the data set using the SQUEEZE routine of PLATON and refined further using the data generated [46]. The final formulas of Tb-*m*-TTFTB, Er-*m*-TTFTB, and Gd-*m*-TTFTB ([Ln_2_(*m*-TTFTB)(*m*-H_2_TTFTB)_0.5_(HCOO)(DMF)]·2DMF·3H_2_O) were calculated from the SQUEEZE results and combined with charge balance, elemental analysis and TGA data. CCDC 1,914,385 (Tb-*m*-TTFTB), 1,914,387 (Er-*m*-TTFTB), and 1,914,384 (Gd-*m*-TTFTB) contain the supplementary crystallographic data for this paper. These data can be obtained free of charge from the Cambridge Crystallographic Data Centre via www.ccdc.cam.ac.uk/data_request/cif (accessed on 8 February 2020) using the accession identifiers CCDC-1,914,384, CCDC-1,914,385 and CCDC-1,914,387, respectively.

### 3.3. Synthesis of m-H_4_TTFTB

*m*-H_4_TTFTB (Figure S19) was prepared according to the reported method [31]. The method is briefly described in the supporting information.

### 3.4. Synthesis of Dy-m-TTFTB, [Dy_2_(m-TTFTB)(m-H_2_TTFTB)_0.5_ (HCOO)(DMF)]·2DMF·3H_2_O

Dy-*m*-TTFTB was prepared according to the reported method [33].

### 3.5. Synthesis of Tb-m-TTFTB, [Tb_2_(m-TTFTB)(m-H_2_TTFTB)_0.5_ (HCOO)(DMF)]·2DMF·3H_2_O

The dissolution of *m*-H_4_TTFTB (0.010 g, 0.015 mmol) in DMF (1 mL) was performed before addition of a solution of TbCl_3_·6H_2_O (0.010 g, ~0.027 mmol) in H_2_O (0.5 mL) followed by the addition of CF_3_COOH (0.17 mL) and chlorobenzene (2 mL). The mixture was heated to 140 °C for 48 h, and then allowed to cool to room temperature. Red rod-like crystals (0.009 g) of Tb-*m*-TTFTB were filtered and washed with DMF and CH_3_COCH_3_ three times. Yield 54% (based on *m*-H_4_TTFTB). Calcd for C_61_H_53_N_3_O_20_S_6_Tb_2_ (Mr = 1658.32 g/mol): C, 44.18; H, 3.22; N, 2.53%. Found: C, 43.58; H, 3.30; N, 2.32%. FT-IR (KBr, cm^−1^): 2929 w, 2360 w, 2341 w, 1676 m, 1653 m, 1635m, 1559 s, 1507 w, 1430 s, 1395 vs, 1254 w, 1163 w, 1083 w, 1000 w, 924 w, 799 m, 763 s, 684 m, 668 s, 657 s, 627 w, 557 w, 442 m.

### 3.6. Synthesis of Er-m-TTFTB, [Er_2_(m-TTFTB)(m-H_2_TTFTB)_0.5_(HCOO)(DMF)]·2DMF·3H_2_O

Er-*m*-TTFTB were synthesized under similar conditions of Tb-*m*-TTFTB except ErCl_3_**·**6H_2_O (0.010 g, ~0.027 mmol). Red rod-like crystals (0.010 g) of Er-*m*-TTFTB were filtered and washed with DMF and CH_3_COCH_3_ three times. Yield 60% (based on *m*-H_4_TTFTB). Calcd for C_61_H_53_N_3_O_20_S_6_Er_2_ (Mr = 1674.99 g/mol): C, 43.74; H, 3.19; N, 2.51%. Found: C, 42.65; H, 3.30; N, 2.43%. FT-IR (KBr, cm^−1^): 3057 w, 2929 w, 2360 w, 2343 w, 1680 m, 1653 m, 1636 m, 1590 m, 1569 m, 1430 s, 1400 vs, 1335 w, 1280 w, 1256 w, 1163 w, 1086 w, 1000 w, 924 w, 800 m, 763 s, 685 m, 668 m, 658 s, 640 w, 625 w, 560 w, 442 m.

### 3.7. Synthesis of Gd-m-TTFTB, [Gd_2_(m-TTFTB)(m-H_2_TTFTB)_0.5_(HCOO)(DMF)]·2DMF·3H_2_O

Gd-*m*-TTFTB. Red rod-like crystals (0.008 g) of Gd-*m*-TTFTB were filtered and washed with DMF and CH_3_COCH_3_ three times. Yield 48% (based on *m*-H_4_TTFTB). Calcd for C_61_H_53_N_3_O_20_S_6_Gd_2_ (Mr = 1654.97 g/mol): C, 44.27; H, 3.23; N, 2.54%. Found: C, 43.82; H, 3.11; N, 2.39%. FT-IR (KBr, cm^−1^): 2929 w, 2360 w, 2341 w, 1676 m, 1632 m, 1590 m, 1560 s,1428 s, 1399 vs, 1255 w, 1084 w, 1000 w, 924 w, 799 m, 763 s, 690 m, 668 m, 657 s, 640 w, 627 w, 556 w, 442 m.

### 3.8. Solid CV

Solid-state cyclic voltammetry measurements were performed in LiBF_4_/CH_3_CN as electrolyte using a Corrtest 4-channel electrochemical workstation and a three-electrode system. The CVs were recorded using a glassy carbon working electrode (3.0 mm diameter), a platinum wire auxiliary electrode, and an Ag wire quasi-reference electrode with the solutions of 0.1 M LiBF_4_ dissolved in distilled CH_3_CN. The sample was mounted on the glassy carbon working electrode by dipping the electrode into a paste made of the powder sample in ethanol. Ferrocene was added as an internal standard upon completion of each experiment. All potentials are reported in milli-Volts (mV) versus the Fc/Fc^+^ couple.

### 3.9. Solid-State Diffuse Reflectance Spectra

The UV-Vis-NIR data were obtained using a Harrick Praying Mantis attachment over the range 200–900 nm. Spectra are reported as the Kubelka–Munk transform.

Kubelka–Munck function:

K = (1 − R)^2^/2R (1)

(Khυ)^1/2^ = B(hυ − *E_g_*) (2)

hυ: photon energy; K: reflection coefficient; B: characteristic constant of material; *E_g_*: band gap.

### 3.10. Electrical Conductivity

The electrical conductivities of the needle-like single crystal samples using the two-probe method were obtained using a Keithley 2400 source meter (Keithley 2400, Tektronix, Beaverton, OR, USA) on CRX-4K Closed Cycle Refrigerator-based Probe Station at room temperature. The single crystal samples were lined in the vertical direction, which were connected by conductive carbon adhesive. All of the current-voltage (I–V) measurements were performed under ambient conditions by sweeping the voltage from −1.5 V to 1.5 V.

## 4. Conclusions

In summary, three redox-active MOFs were constructed by lanthanide metal ions (Tb^3+^, Er^3+^, and Gd^3+^) and *m*-TTFTB. These MOFs showed similar three-dimensional lattice with a dense stacking. It can be concluded that compared with H_4_TTFTB, the assembly of *m*-H_4_TTFTB tends to form a structure with almost no porosity. Magnetic study revealed that Dy-*m*-TTFTB and Er-*m*-TTFTB possess slow relaxation of the magnetization. In all, even though *m*-TTFTB-based MOFs seem not to be a good porous material, these dense stacking structures may enable them as good candidates for the study of charge transfer, electrical conductivity, and magnetic properties. Further studies focusing on the functional assembly of *m*-H_4_TTFTB and other metal building blocks are currently in progress in our group.

## Figures and Tables

**Table 1:** Crystal and structure refinement parameters for Tb-*m*-TTFTB, Er-*m*-TTFTB, and Gd-*m*-TTFTB.

	Tb-*m*-TTFTB	Er-*m*-TTFTB	Gd-*m*-TTFTB
CCDC number	1,914,385	1,914,387	1,914,384
Empirical formula	C_61_H_53_N_3_O_20_S_6_Tb_2_	C_61_H_53_N_3_O_20_S_6_Er_2_	C_61_H_53_N_3_O_20_S_6_Gd_2_
Formula weight	1658.32	1674.99	1654.97
Temperature (K)	153	153	153
Crystal system	Triclinic	Triclinic	Triclinic
Wavelength (Å)	0.71073	0.71073	0.71073
Space group	*P*-1 (No. 2)	*P*-1 (No. 2)	*P*-1 (No. 2)
*a* (Å)	15.5977 (10)	15.371 (16)	15.5779 (7)
*b* (Å)	15.696 (1)	15.15 (3)	15.7382 (7)
*c* (Å)	16.6914 (11)	16.441 (18)	16.7290 (7)
*α* (°)	106.077 (1)	105.29 (4)	106.761 (1)
*β* (°)	114.426 (1)	115.493 (15)	114.410 (1)
*γ* (°)	104.542 (1)	104.25 (3)	104.065 (2)
Volume (Å^3^)	3248.0 (4)	3036 (8)	3253.0 (3)
Z	2	2	2
d (g·cm^−3^)	1.491	1.613	1.485
F(000)	1432	1444	1428
Absorption coefficient, *μ*/mm^−1^	2.411	3.013	2.272
Reflections total	29,069	28,223	29,492
Reflections independent	14,332	13,867	15,158
*θ*-range (°)	1.5–27.3	1.5–27.9	2.1–27.7
*R*_int_	0.029	0.027	0.025
*R*_1_, ^(a)^ *wR*_2_ (I ≥ 2σ(I)) ^(b)^	0.0327/0.1031	0.0445/0.1375	0.0295/0.0775
*R*_1_, *wR*_2_ (all data)	0.0424/0.1314	0.0546/0.1614	0.0353/0.0805
GOF	1.05	1.10	1.06
Largest diff. peak and hole (eÅ^−3^)	1.66/−1.12	2.70/−1.71	2.49/−1.15

^(a)^ *R*_1_ = Σ||F_o_| − |F_c_||/Σ|F_o_|; ^(b)^ w*R*_2_ = [Σw(F_o_^2^ – F_c_^2^)^2^/Σw(F_o_^2^)^2^]^1/2^.

## Data Availability

CCDC 1914384, 1914385, and 1914387 contains the supplementary crystallographic data for this paper. These data are provided free of charge by the joint Cambridge Crystallographic Data Centre www.ccdc.cam.ac.uk/structures (accessed on 8 February 2020) using the accession identifiers CCDC-1,914,384, CCDC-1,914,385 and CCDC-1,914,387, respectively. All other data can be obtained from the authors on request.

## Supplementary Materials

The following supporting information can be downloaded at: https://www.mdpi.com/article/10.3390/molecules27134052/s1, Table S1: Selected bond lengths (Å) and angles (°) of Tb-*m*-TTFTB, Er-*m*-TTFTB, and Gd-*m*-TTFTB; Table S2: The shape parameters of Tb-*m*-TTFTB, Er-*m*-TTFTB, and Gd-*m*-TTFTB single crystals used for the calculating of electrical conductivity; Figure S1: The crystal photos of Tb-*m*-TTFTB (**a**), Er-*m*-TTFTB (**b**), and Gd-*m*-TTFTB (**c**); Figure S2: The asymmetric units of Tb-*m*-TTFTB. Displacement ellipsoids are drawn at the 50% probability level; Figure S3: The coordination environment of L1 (**top**) and L2 (**down**) in Tb-*m*-TTFTB; Figure S4. The three-dimensional structures of Tb-*m*-TTFTB along *a*, *b*, and *c* directions. Figure S5: The TGA plots of Tb-*m*-TTFTB, Er-*m*-TTFTB, and Gd-*m*-TTFTB under an N_2_ atmosphere; Figure S6: Solid state cyclic voltammograms of Er-*m*-TTFTB performed over three consecutive cycles (**a**) and at different scan rates (**b**). The experiments were conducted in 0.1 M LiBF_4_ in CH_3_CN electrolyte; Figure S7: Solid-state cyclic voltammograms of Gd-*m*-TTFTB were performed over three consecutive cycles (**a**) and at different scan rates (**b**). The experiments were conducted in 0.1 M LiBF_4_ in CH_3_CN electrolyte; Figure S8: The picture of the single crystal of Tb-*m*-TTFTB and the electric device used for the measurement of electrical conductivity; Figure S9: I–V curve of Tb-*m*-TTFTB; Figure S10: I–V curve of Er-*m*-TTFTB; Figure S11: I–V curve of Gd-*m*-TTFTB; Figure S12: Three kinds of exchange interactions in one-dimensional chain of Gd-*m*-TTFTB; Figure S13: Temperature dependence of the *χ*_M_*T* for Gd-*m*-TTFTB measured in a 1000 Oe field. The red line is the simulation of two isolated Gd ions. The blue line is the simulation of Gd2 cluster only existing magnetic coupling; Figure S14: The field-dependent magnetizations from 0 to 70 kOe at 1.8 K for Tb-*m*-TTFTB; Figure S15: The field-dependent magnetizations from 0 to 70 kOe at 1.8 K for Dy-*m*-TTFTB; Figure S16: The field-dependent magnetizations from 0 to 70 kOe at 1.8 K for Er-*m*-TTFTB; Figure S17: (**a**) Temperature-dependent in-phase χ′ and out-of-phase χ″ ac susceptibility signals for Tb-*m*-TTFTB at the indicated frequencies under zero dc field. (**b**) Temperature-dependent in-phase χ′ and out-of-phase χ″ ac susceptibility signals for Tb-*m*-TTFTB at the indicated frequencies under 1000 dc field; Figure S18: (**a**) Temperature-dependent in-phase χ′ and out-of-phase χ″ ac susceptibility signals for Er-*m*-TTFTB at the indicated frequencies under zero dc field. (**b**) Temperature-dependent in-phase χ′ and out-of-phase χ″ ac susceptibility signals for Er-*m*-TTFTB at the indicated frequencies under 1000 dc field; Figure S19. The synthesis route of *m*-H_4_TTFTB.

## Sample Availability

Samples of the compounds are available from the authors.
