# Supplementary material for: Redox-Active Metal-Organic Frameworks with Three-Dimensional Lattice Containing the m-Tetrathiafulvalene-Tetrabenzoate
Source: Molecules. 2022 Jun 23;27(13):4052. doi: 10.3390/molecules27134052 (PMC9268712; doi:10.3390/molecules27134052)
Supplement: Supplementary file 1 [file molecules-27-04052-s001.zip › molecules-1755985-supplementary/molecules-1755985-supplementarydone.pdf]

molecules

Supporting Material

Redox-Active Metal-Organic Frameworks with Three-Dimensional Lattice Containing the *m*-Tetrathiafulvalene-Tetrabenzoate

Hongrui Huang [1], Zhi-Mei Yang [2], Xiao-Cheng Zhou [2] and Gen Zhang [1,*], Jian Su [1,2,*]

1 School of Chemistry and Chemical Engineering, Nanjing University of Science and Technology, Nanjing 210094, China; hhrvinc@njust.edu.cn (H.H.)
2 State Key Laboratory of Coordination Chemistry, Nanjing University, Nanjing 210023, China; dg21240145@smail.nju.edu.cn (Z.-M.Y.); mg20240142@smail.nju.edu.cn (X.-C.Z.)
* Correspondence: zhanggen@njust.edu.cn (G.Z.); sujian@njust.edu.cn (J.S.)

Table S1. Selected bond lengths (Å) and angles (°) of Tb-*m*-TTFTB, Er-*m*-TTFTB, and Gd-*m*-TTFTB.

	**Tb-*m*-TTFTB**	**Er-*m*-TTFTB**	**Gd-*m*-TTFTB**
RE(1)-O(1)^{i}	2.324 (3)	2.257 (5)	2.356 (2)
RE(1)-O(3)^{ii}	2.320 (3)	2.241 (5)	2.3338 (19)
RE(1)-O(4)	2.296 (3)	2.210 (5)	2.310 (2)
RE(1)-O(5)^{iii}	2.314 (3)	2.259 (6)	2.341 (2)
RE(1)-O(6)^{iv}	2.317 (3)	2.219 (5)	2.314 (2)
RE(1)-O(9)	2.400 (3)	2.332 (7)	2.416 (2)
RE(1)-O(15)^{v}	2.478 (3)	2.368 (5)	2.504 (2)
RE(2)-O(2)^{iv}	2.299 (3)	2.246 (5)	2.309 (2)
RE(2)-O(7)	2.266 (3)	2.186 (4)	2.272 (2)
RE(2)-O(8)^{vi}	2.339 (3)	2.261 (5)	2.348 (2)
RE(2)-O(11)^{vii}	2.331 (3)	2.249 (5)	2.360 (2)
RE(2)-O(12)^{viii}	2.325 (3)	2.269 (5)	2.322 (3)
RE(2)-O(13)	2.332 (4)	2.249 (6)	2.355 (3)
RE(2)-O(14)	2.406 (3)	2.309 (5)	2.425 (2)
O(1)^{i}-RE(1)-O(9)	72.65 (12)	72.52 (18)	71.89 (8)
O(1)^{i}-RE(1)-O(15)^{v}	98.81 (12)	95.8 (2)	100.14 (8)
O(3)^{ii}-RE(1)-O(1)^{i}	144.63 (12)	144.82 (18)	143.45 (8)
O(3)^{ii}-RE(1)-O(5)^{iii}	74.80 (12)	74.7 (2)	74.60 (8)
O(3)^{ii}-RE(1)-O(9)	73.84 (12)	74.05 (18)	73.81 (8)
O(3)^{ii}-RE(1)-O(15)^{v}	81.16 (11)	85.02 (19)	80.48 (7)
O(4)-RE(1)-O(1)^{i}	81.67 (12)	81.34 (19)	82.59 (8)
O(4)-RE(1)-O(3)^{ii}	123.15 (12)	123.40 (17)	122.55 (8)
O(4)-RE(1)-O(5)^{iii}	76.74 (12)	76.2 (2)	76.39 (8)
O(4)-RE(1)-O(6)^{iv}	76.99 (12)	77.3 (2)	76.43 (8)
O(4)-RE(1)-O(9)	146.22 (11)	145.6 (2)	146.70 (8)
O(4)-RE(1)-O(15)^{v}	134.96 (11)	132.16 (18)	135.44 (7)
O(5)^{iii}-RE(1)-O(1)^{i}	139.67 (12)	139.74 (19)	141.24 (8)
O(5)^{iii}-RE(1)-O(9)	136.77 (12)	137.8 (2)	136.59 (8)
O(5)^{iii}-RE(1)-O(15)^{v}	74.54 (12)	76.2 (2)	74.43 (9)
O(6)^{iv}-RE(1)-O(1)^{i}	82.45 (12)	83.3 (2)	82.10 (8)
O(6)^{iv}-RE(1)-O(3)^{ii}	79.93 (11)	79.24 (19)	79.43 (7)
O(6)^{iv}-RE(1)-O(5)^{iii}	124.08 (13)	122.6 (2)	122.80 (9)
O(6)^{iv}-RE(1)-O(9)	78.18 (12)	77.9 (2)	79.12 (9)
O(6)^{iv}-RE(1)-O(15)^{v}	148.02 (12)	150.2 (2)	148.12 (8)
O(9)-RE(1)-O(15)^{v}	71.89 (11)	73.6 (2)	71.63 (8)
O(2)^{iv}-RE(2)-O(8)^{vi}	130.28 (13)	131.43 (19)	130.60 (9)
O(2)^{iv}-RE(2)-O(11)^{vii}	151.92 (13)	151.09 (18)	151.14 (10)
O(2)^{iv}-RE(2)-O(12)^{viii}	76.01 (12)	78.53 (17)	76.29 (9)
O(2)^{iv}-RE(2)-O(13)	78.15 (14)	76.2 (2)	77.09 (10)
O(2)^{iv}-RE(2)-O(14)	76.84 (11)	76.97 (19)	76.96 (7)
O(7)-RE(2)-O(2)^{iv}	88.36 (12)	87.04 (19)	88.49 (8)
O(7)-RE(2)-O(8)^{vi}	125.80 (12)	125.80 (17)	125.96 (9)
O(7)-RE(2)-O(11)^{vii}	80.38 (12)	81.1 (2)	80.38 (9)
O(7)-RE(2)-O(12)^{viii}	78.52 (13)	76.9 (2)	78.41 (10)
O(7)-RE(2)-O(13)	83.09 (13)	83.1 (2)	82.35 (10)
O(7)-RE(2)-O(14)	159.06 (12)	158.10 (16)	158.46 (9)
O(8)^{vi}-RE(2)-O(11)^{vii}	76.00 (12)	75.8 (2)	76.16 (9)
O(8)^{vi}-RE(2)-O(13)	133.95 (13)	135.0 (2)	134.62 (10)
O(8)^{vi}-RE(2)-O(14)	75.06 (11)	76.06 (17)	75.38 (8)
O(11)^{vii}-RE(2)-O(14)	105.91 (12)	106.0 (2)	104.87 (8)
O(12)^{viii}-RE(2)-O(8)^{vi}	77.26 (12)	74.7 (2)	77.76 (9)
O(12)^{viii}-RE(2)-O(11)^{vii}	125.79 (12)	124.33 (18)	126.35 (10)
O(12)^{viii}-RE(2)-O(13)	148.52 (14)	150.0 (2)	147.36 (10)
O(12)^{viii}-RE(2)-O(14)	111.38 (12)	111.13 (19)	112.61 (9)
O(13)-RE(2)-O(11)^{vii}	75.03 (13)	76.3 (2)	75.10 (10)

O(13)-RE(2)-O(14)	79.46 (13)	78.62 (19)	79.00 (9)

Symmetry codes: (i) -x+2, -y+2, -z+2; (ii) -x+2, -y+1, -z+2; (iii) x+1, y, z+1; (iv) -x+1, -y+1, -z+1; (v) x+1, y+1, z+1; (vi) -x+1, -y, -z+1; (vii) x-1, y-1, z; (viii) -x+2, -y+1, -z+1.

Table S2. The shape parameters of Tb-*m*-TTFTB, Er-*m*-TTFTB, and Gd-*m*-TTFTB single crystals used for the calculating of electrical conductivity.

	**Length (cm)**	**Width (cm)**	**Thickness (cm)**	**Cross-sectional Area (cm^2)**	**Electrical Conductance (G)**	**Electrical Conductivity (S/cm)**
Tb-*m*-TTFTB	0.028	0.006	0.006	3.6e-5	7.0e-10	5.4e-7
Er-*m*-TTFTB	0.034	0.004	0.004	1.6e-5	4.5e-10	9.6e-7
Gd-*m*-TTFTB	0.007	0.005	0.005	2.5e-5	3.7e-10	1.0e-7

$$\sigma = \mathrm{G}\frac{L}{A}$$

Electrical conductivity, σ, measures a material's ability to conduct electrical current. Measuring σ typically requires incorporating the material of interest into an electronic device, typically a resistor, and measuring the electrical conductance (G), length (L), and cross-sectional area (A) of the conduction channel.

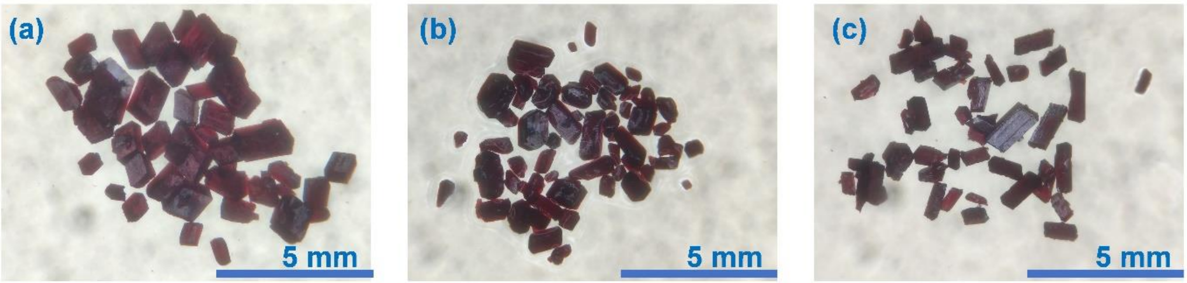

Figure S1. The crystal photos of Tb-*m*-TTFTB (**a**), Er-*m*-TTFTB (**b**), and Gd-*m*-TTFTB (**c**).

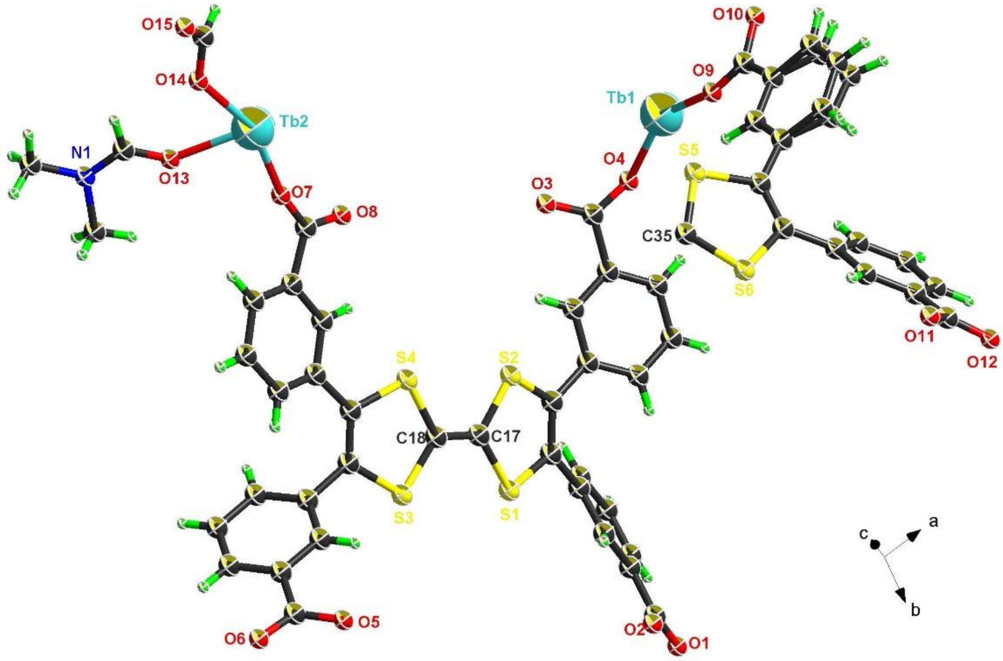

Figure S2. The asymmetric units of Tb-*m*-TTFTB. Displacement ellipsoids are drawn at the 50% probability level.

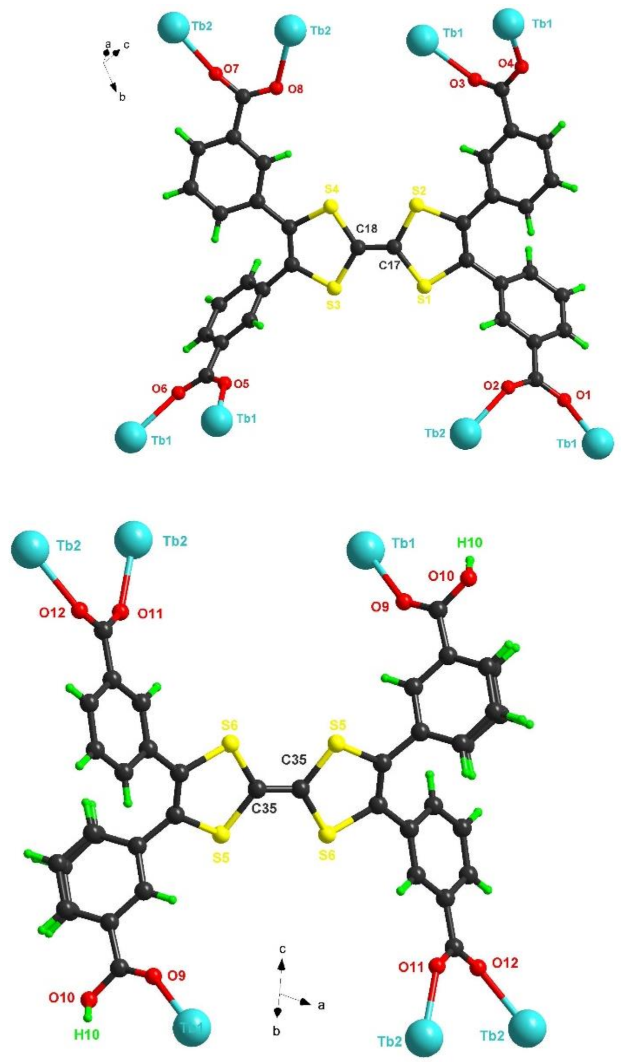

Figure S3. The coordination environment of L1 (**top**) and L2 (**down**) in Tb-*m*-TTFTB.

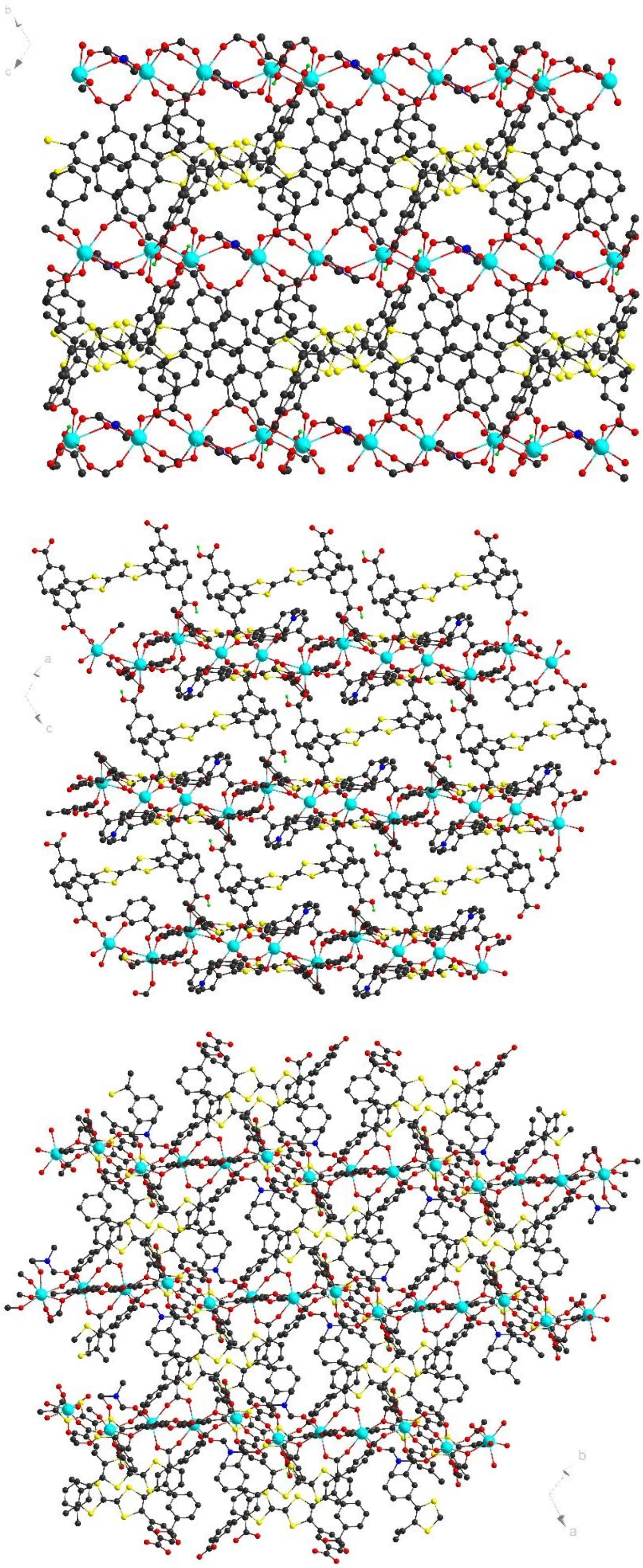

Figure S4. The three-dimensional structures of Tb-*m*-TTFTB along *a*, *b*, and *c* directions.

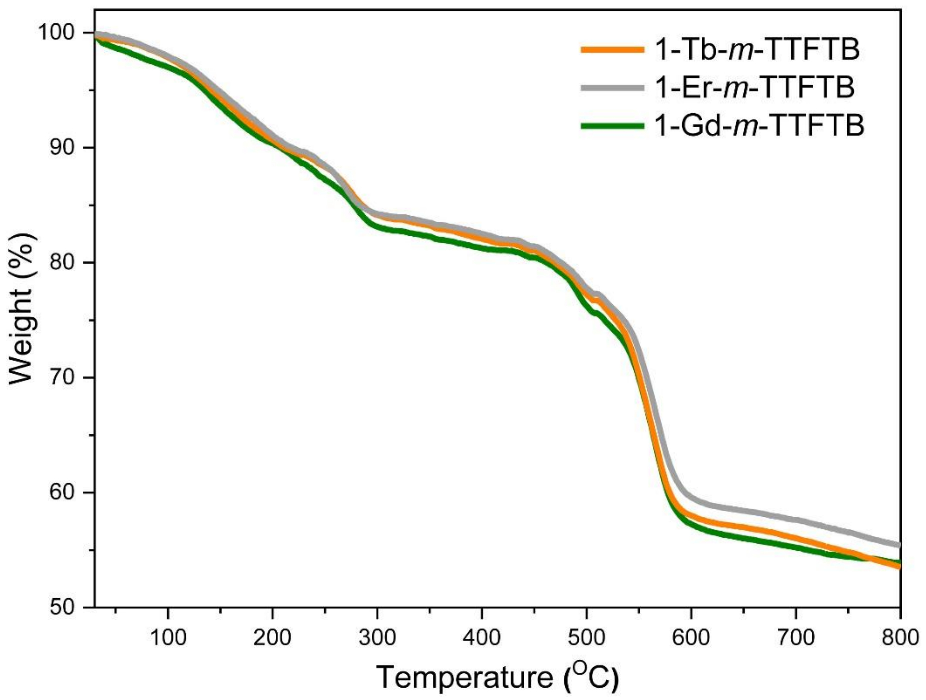

Figure S5. The TGA plots of Tb-*m*-TTFTB, Er-*m*-TTFTB, and Gd-*m*-TTFTB under an N_2 atmosphere.

Thermogravimetric analyses of Tb-*m*-TTFTB, Er-*m*-TTFTB, and Gd-*m*-TTFTB were conducted from room temperature to 800 °C under N_2 atmosphere. These four compounds exhibit similar thermal behavior. For Gd-*m*-TTFTB, the weight losses about 3.4% at 110 °C (calcd 3.3% for three H_2O), 13.7% at 310 °C (calcd 13.3% for two free DMF and one coordinated DMF) correspond to the release of three DMF. (Tb-*m*-TTFTB, ~2.8%, calcd 3.3% at 110 °C, 13.3%, calcd 13.2% at 310 °C; Er-*m*-TTFTB, ~2.6%, calcd 3.2% at 110 °C, ~13.2%, calcd 13.1% at 310 °C). A plateau up to ca. 450 °C was followed, implying that compounds Tb-*m*-TTFTB, Er-*m*-TTFTB, and Gd-*m*-TTFTB were stable up to 450 °C. After the plateau, Tb-*m*-TTFTB, Er-*m*-TTFTB, and Gd-*m*-TTFTB began to decompose.

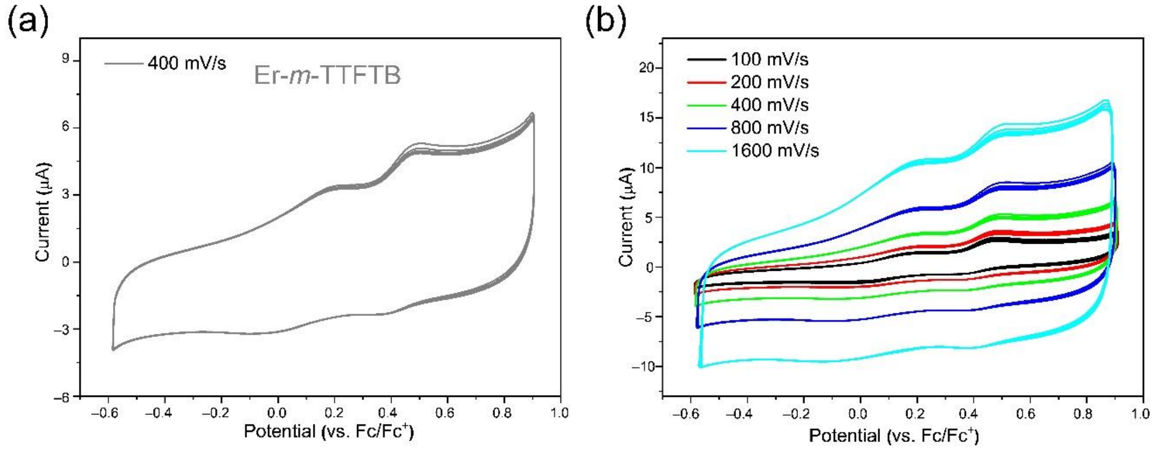

Figure S6. Solid state cyclic voltammograms of Er-*m*-TTFTB performed over three consecutive cycles (**a**) and at different scan rates (**b**). The experiments were conducted in 0.1 M $LiBF_4$ in CH_3CN electrolyte.

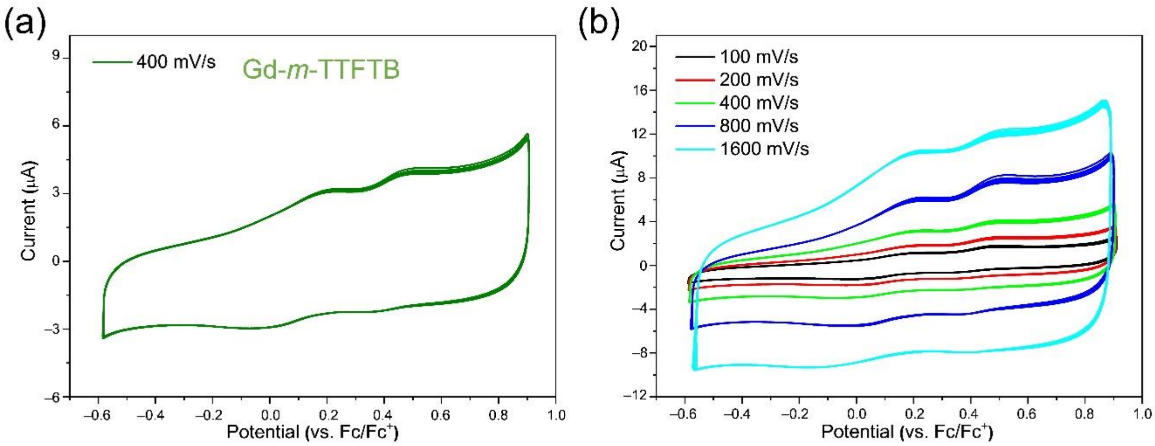

Figure S7. Solid state cyclic voltammograms of Gd-*m*-TTFTB performed over three consecutive cycles (a) and at different scan rates (b). The experiments were conducted in 0.1 M $LiBF_4$ in CH_3CN electrolyte.

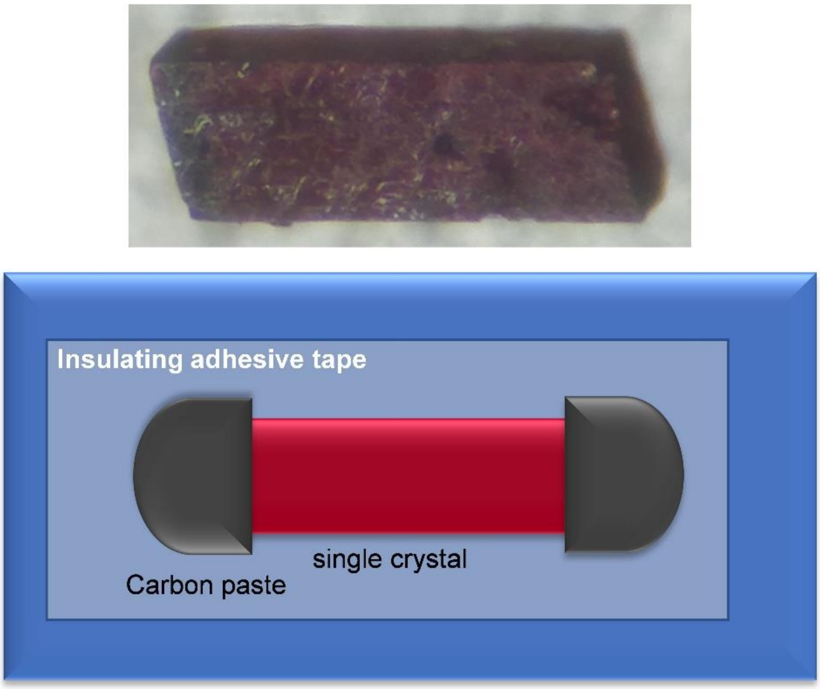

Figure S8. The picture of the single crystal of Tb-*m*-TTFTB and the electric device used for the measurement of electrical conductivity.

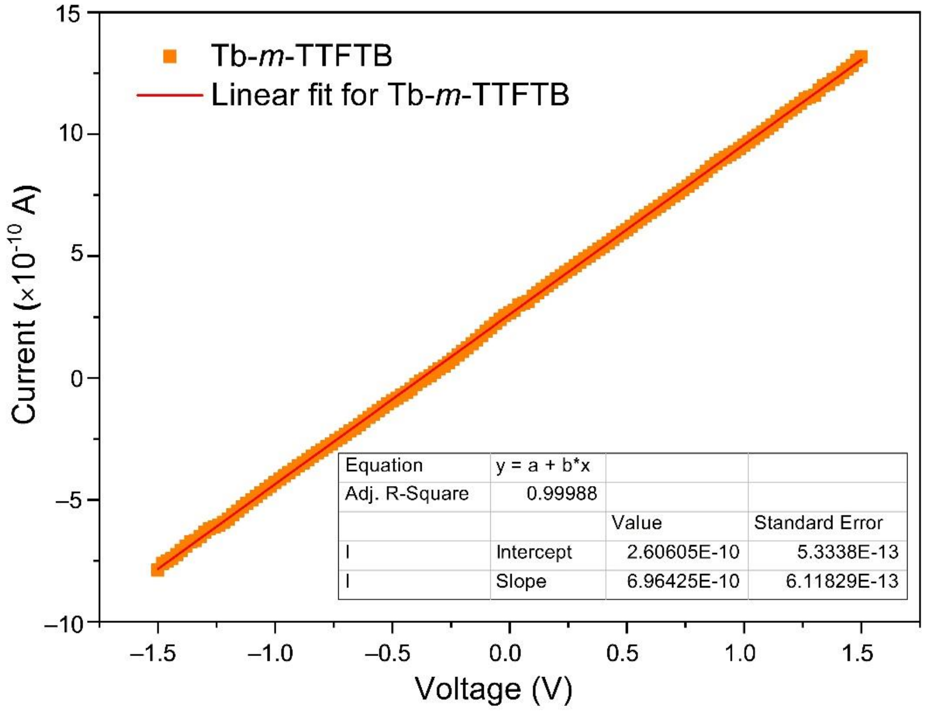

Figure S9. I–V curve of Tb-*m*-TTFTB.

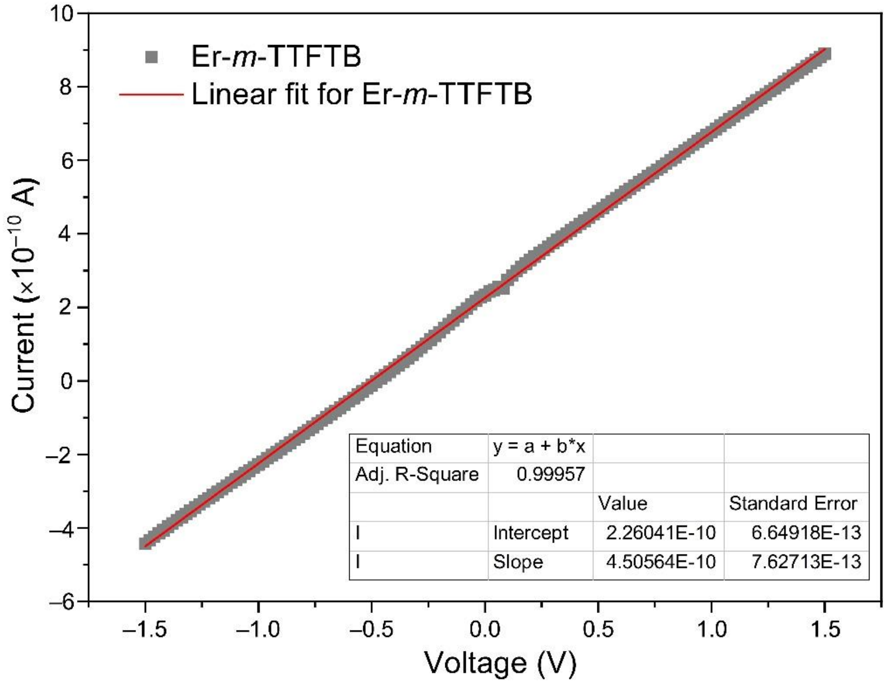

Figure S10. I–V curve of Er-*m*-TTFTB.

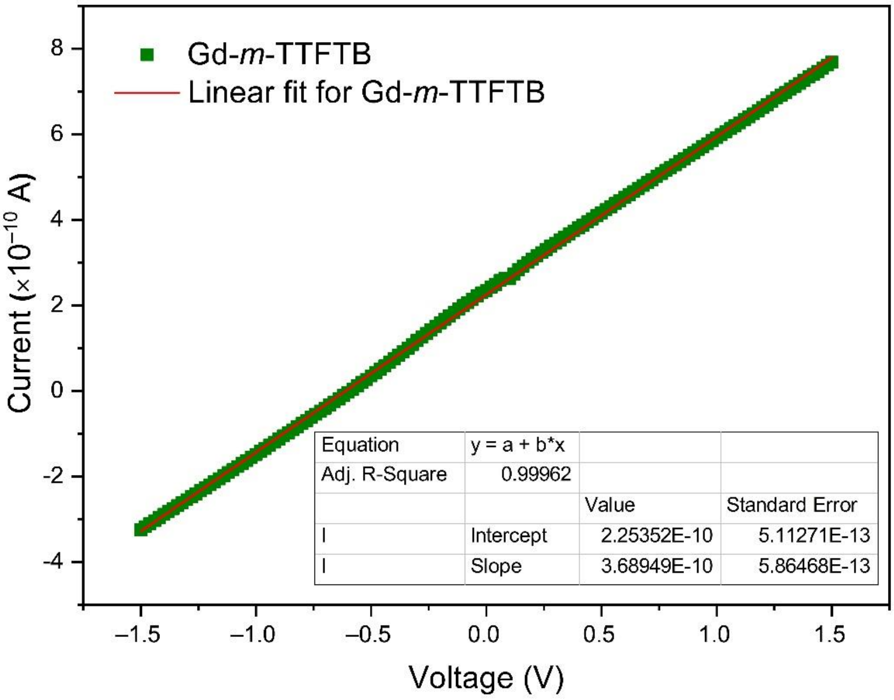

Equation	y = a + b*x		
Adj. R-Square	0.99962		
		Value	Standard Error
I	Intercept	2.25352E-10	5.11271E-13
I	Slope	3.68949E-10	5.86468E-13

Figure S11. I–V curve of Gd-*m*-TTFTB.

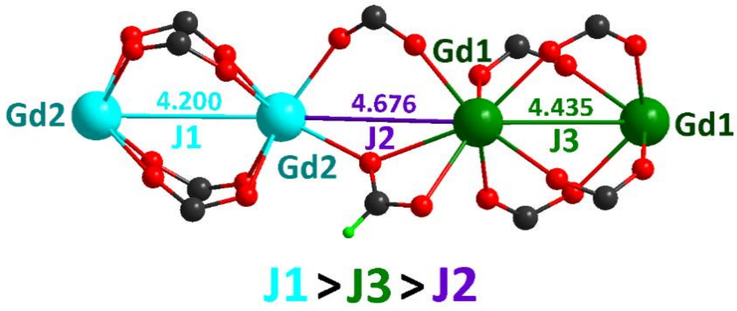

Figure S12. Three kinds of exchange interactions in one-dimensional chain of Gd-*m*-TTFTB.

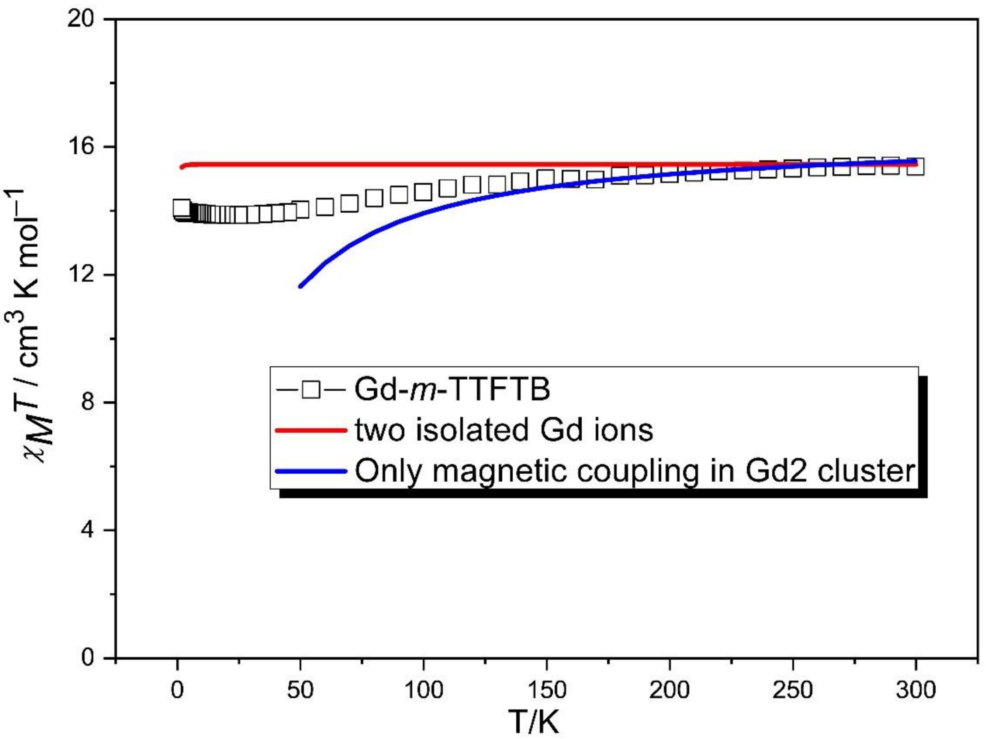

Figure S13. Temperature dependence of the $\chi_M T$ for Gd-*m*-TTFTB measured in a 1000 Oe field. The red line is the simulation of two isolated Gd ions. The blue line is the simulation of Gd2 cluster only existing magnetic coupling.

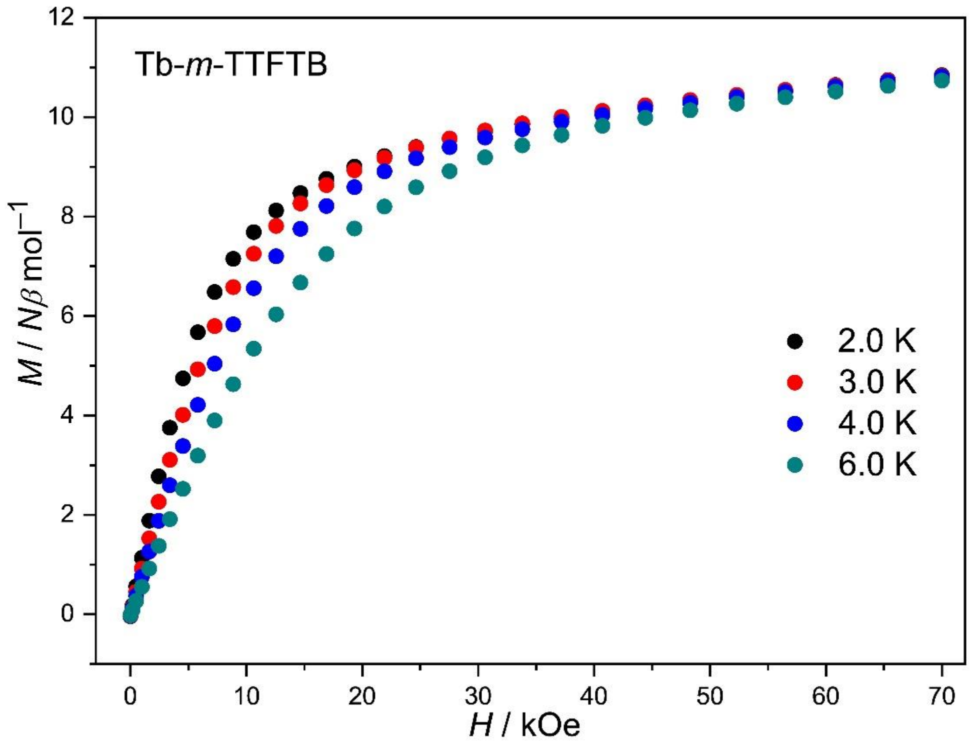

Figure S14. The field-dependent magnetizations from 0 to 70 kOe at 1.8 K for Tb-*m*-TTFTB.

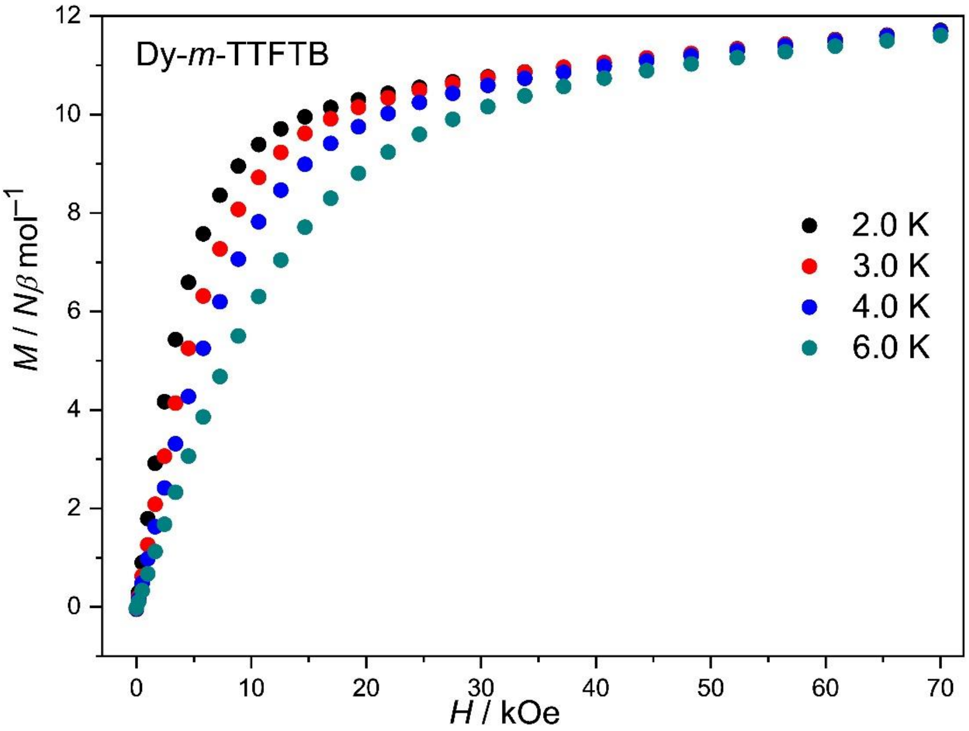

Figure S15. The field-dependent magnetizations from 0 to 70 kOe at 1.8 K for Dy-*m*-TTFTB.

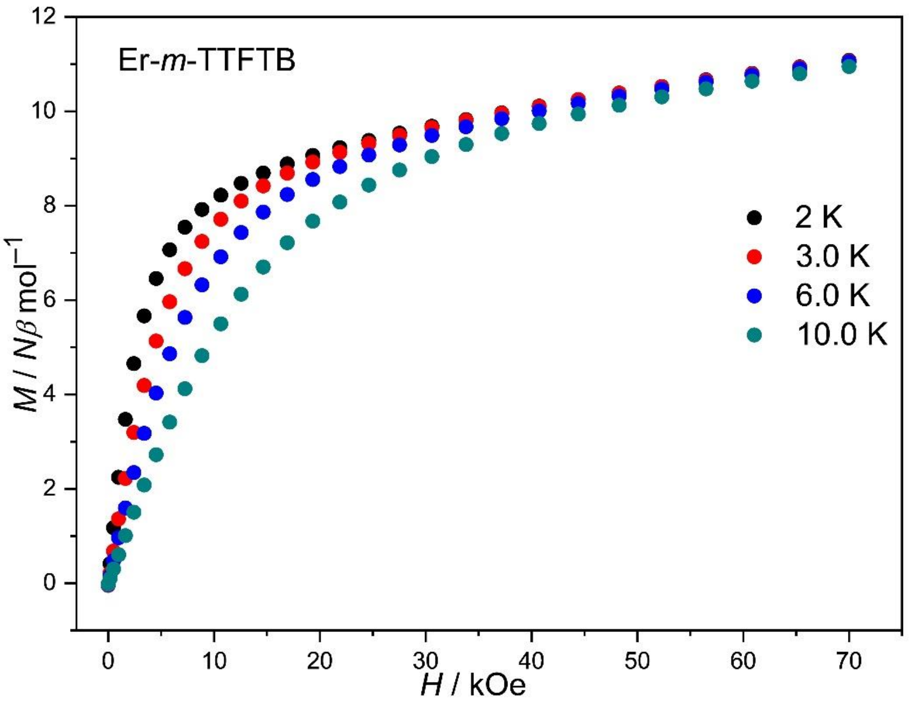

Figure S16. The field-dependent magnetizations from 0 to 70 kOe at 1.8 K for Er-*m*-TTFTB.

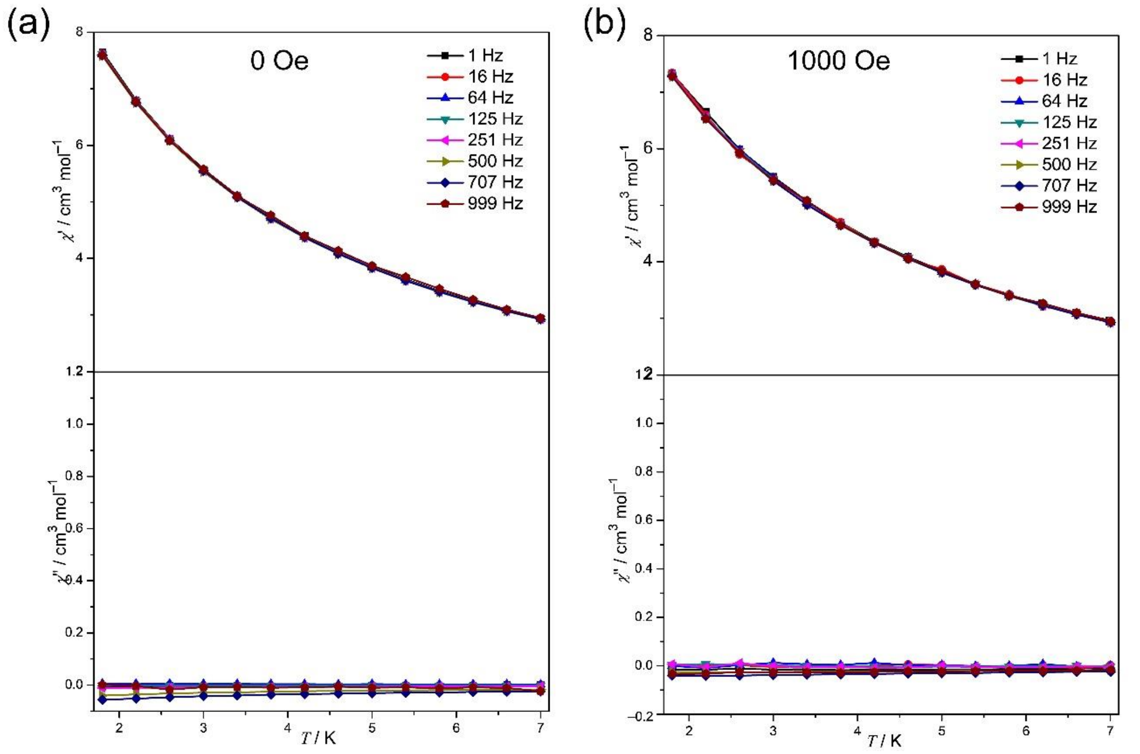

Figure S17. (a) Temperature-dependent in-phase χ′ and out-of-phase χ″ ac susceptibility signals for Tb-*m*-TTFTB at the indicated frequencies under zero dc field. (b) Temperature-dependent in-phase χ′ and out-of-phase χ″ ac susceptibility signals for Tb-*m*-TTFTB at the indicated frequencies under 1000 dc field.

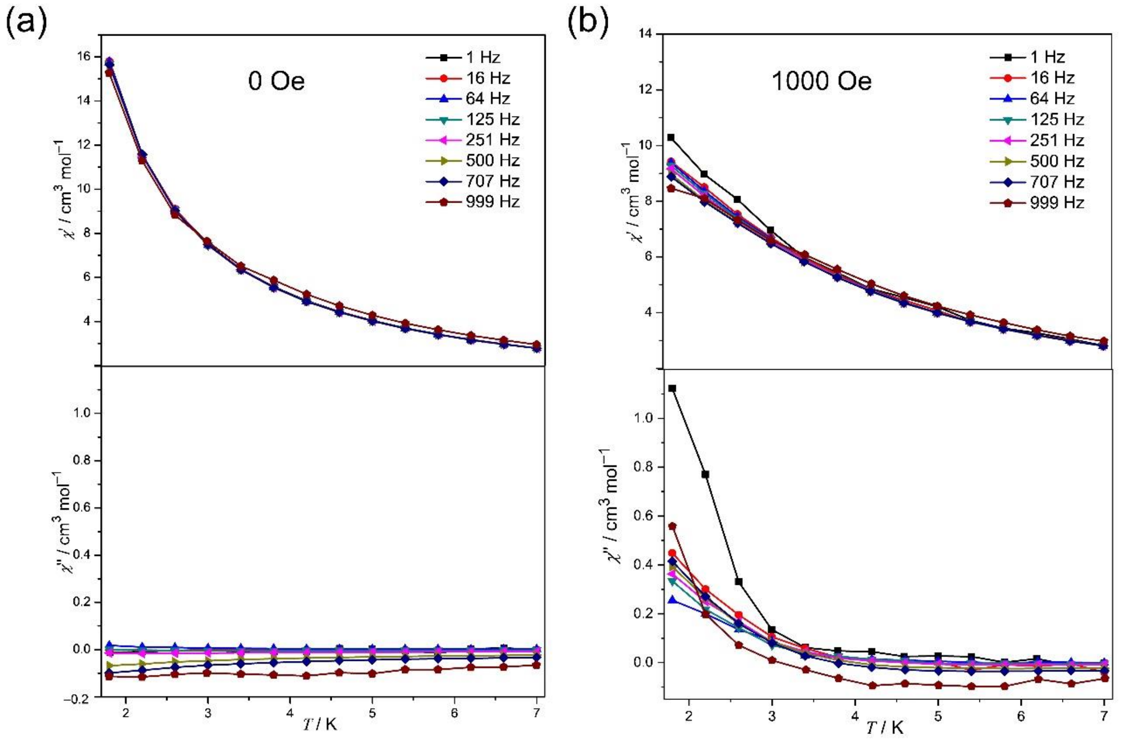

Figure S18. (a) Temperature-dependent in-phase χ′ and out-of-phase χ″ ac susceptibility signals for Er-*m*-TTFTB at the indicated frequencies under zero dc field. (b) Temperature-dependent in-phase χ′ and out-of-phase χ″ ac susceptibility signals for Er-*m*-TTFTB at the indicated frequencies under 1000 dc field.

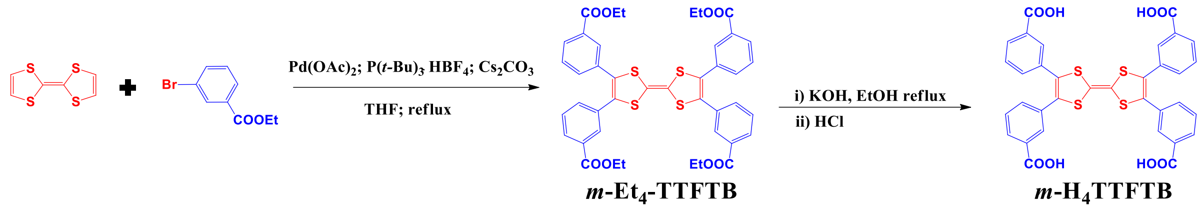

Figure S19. The synthesis route of *m*-H_4TTFTB.

Pd(OAc)$_2$ (410 mg, 1.83 mmol), P(*t*-Bu)$_3$HBF$_4$ (1.60 g, 5.51 mmol), and Cs_2CO_3 (12 g, 36.83 mmol) were placed in a 250-mL reaction flask under nitrogen. The degassed THF (100 mL) was added and the mixture was stirred for 30 min with heating at 60 °C. Tetrathiafulvalene (1.5 g, 7.34 mmol) and ethyl *m*-bromobenzoate (9.47 g, 41.34 mmol) was added. The mixture was heated at reflux for 72 h. The product was concentrated in vacuo to solid. The organic compounds were extracted with dichloromethane (DCM) through filtration and concentrated in vacuo. Chromatographic purification on silica gel by using petroleum ether/DCM (1:3) as an eluent afforded *m*-Et$_4$-TTFTB (2.2 g, 2.76 mmol, 37.6%) as an orange solid.

m-Et$_4$-TTFTB (4.10 g, 5.14 mmol) and THF (150 mL)/MeOH (150 mL) were placed in a 500-mL reaction flask under air. A solution of KOH (4.0 g, 71.28 mmol) dissolved in 30 mL water was added and the mixture was stirred overnight with heating at 90 °C. The product was concentrated in vacuo to red oil or solid. Water (60 mL) was added to dissolve the red solid. HCl was added to adjust the solution to pH 2. The red solid was obtained by centrifugation and washed three times with water. After vacuum drying, *m*-H_4TTFTB (3.22 g, 4.70 mmol, 81%) was afforded as a red solid.
